# Supplementary material for: Plastomes of Garcinia mangostana L. and Comparative Analysis with Other Garcinia Species
Source: Plants (Basel). 2023 Feb 17;12(4):930. doi: 10.3390/plants12040930 (PMC9966718; doi:10.3390/plants12040930)
Supplement: Supplementary file 1 [file plants-12-00930-s001.zip › Supplementary- DataS1.pdf]

Supplementary Data S1. Adjusted ORF of plastome genes from different *Garcinia* species.

>G. anomala_rps19
GTGTCACGTTCACTAAAAAAAAAATCCTTTTGTAGCAATTCATTTATTAAGAAAAATAAAT
AAGCTTAACACAAAAGAAGAAAAAGAAATAATAGTAACATGGTCACGAGCATCAACCATT
ATACCTACAATGATTGGCCATACTATTGCTATCCATAATGGAAAAGAACATTTACCTATT
TATATAACAGATCGTATGGTGGGTCATAAATTGGGCGAATTTTCCCCTACTCTAAATTTC
CGGGGACATGCAAAAAATGATAATAAATCTCGTCGATAA

>G. anomala_cemA
ATGAGGCGGGTTCATTTAAAATACGAAAAAATGAAAAAAAAAAACCTTATTTCCCTTCTA
TATTTTATATCTATAATATTGTTGCCCTGGTGGATCTCTTTTTCGTTTAATAAAAGTGTG
GAATCTTGGGTTATTAATTGGTGGAATACTAGTCAATCTGAAATTTTTTTAAATGCTATT
CAAGAAAAGAGTAGTTTAGAAAAATTCATAGAATTAGAGGAACTCTTACTCTTGGACGAA
ATGATAAAGGAATGCCCGGAAACACATCTGCAAAAGTTTATTATCGTAATCCACAAAGAA
ACGATCAAATTGCTCAAAATGTACAATGAGGAGCGTATCTATACTATTTTGCACTTCTCA
ACCAATATAATCTGTTTCGTTATTCTAAGCGGTTATTCGATTCTAGGTAATGAAGAACTT
TTTCTTCTTAATTCTTGGGTTCAAGAATTTCTATATAACCTAAGTGATACAATAAAAGCT
TTTTTTATTCTTTTATTAACCGATTTATGTATAGGATTCCATTCACCCCACGGCTGGGAA
CTACTGATTGGTTCTGTCTACAAGGATTTTGGATTTACTTATAACGATCAAATCATATCT
GGACTTGTTTCCACTTTTCCAGTAATTATCGATACAATTTTAAAATATTGGATCTTCCAT
TATTTAAATCGTGTATCTCCGTCACTTGTAGTGATTTATCATTCAATGAATGACTGA

>G. anomala_ndhA
ATGAATATAACAGACATAATTGATATAATAGAAATTAATTCTTTTTCTAGATTGAGATTG
GAATCTTTAAACGAGATCTATGGAATTCTATGGATGCTTTTCCCTATTTTTATTCTGATA
TTGGGAATCATGATAGGTGTATTAGTAATTGTGTGGTTAGAAAGAGAAATATCTGCAGGG
GTGCAACAACGTATTGGACCTGAGTATGCCGGTCCTTTTGGAGTTCTTCAAGCGCTAGCG
GACGGGACAAAATTACTTTTCAAAGAGAATCTTTTTCCGTCTCGAGGAGATACTCGTTTA
TTCAGTCTTGGACCAGCCATAGCAGTCATATCAACTCTATTAAGCTATTCCGTAATTCCT
TTTAGCTATCACCTTGTTTTAGCTGATCTAACTATTGGTGTTTTTTTATGGATTGCCATT
TCAAGTATTGCCCCTATTGGCCTTCTTATGTCAGGGTATGGATCAAACAATAAATATTCC
TTTTTAGGTGGTCTACGAGCTGCTGCTCAATCAATTAGTTATGAAATACCATTAACTCTC
TGCGTATTATCCATATCTCTATTATCTAACAGTTCAAGTACAGTTGATATAGTTGAAGCA
CAATCAAAATATGGTTTTTGGGGGTGGAATTTTTGGCGTCAACCTATAGGGTTTATCATT
TTTTTAATTTCTTCCTTAGCGGAATGTGAGAGATTGCCCTTTGATTTACCAGAAGCAGAA
GAAGAATTAGTAGCAGGTTATCAAACCGAATATTCGGGTATTAAATTTGGTTTATTTTAT
GTTGCTTCCTATCTAAACTTATTAGTTTCGTCATTATTTGTAACAGTTCTTTACTTGGGT
GGTTGGAATATCTCTATTCCATTCCTTCCTGAATTTTTTGAGCTAACTAAAGTAAATGGA
GTCTGTGGAACAATAATGGGGATCTTTATTACATTAGTTAAAAGTTATTTGTTCTTGTTC
ATTTCTATCACAATAAGATGGACTTTACCTAGACTAAGAATGGATCAACTATTAAATCTT
GGATGGAAATTTCTTTTACCTATATCTCTTGGTAATTTAGTATTAACAACTTCTTTCCAA
TTACTTTCACTCTAA

>G. anomala_ndhD
ACGAATTCTTTTCCTTGGTTAACATTATTTGTAGTTTTACCGATATCCGCGGGTTTTTTA
ATTTTCTTTTTTCCTCATAGAGGTAATAAGGTAATTCGCTGGTATACTATTAGTATTTGT
GTTTTAGAGCTCCTTTTAATGACTTATATATTTTTGTATTATTTTAAATTGGATGATCCA
TTAATACAATTAACAGAGAGTTCTAAATGGATCAATTTTTTGAATTTTTACTGGAGATTG

GGAATAGATGGGATCTCTTTAGGACCCATTTTTTTGACCGGATTTATCACTACTTTAGCT
ACTTTAGCGGCTCGGCCAGTTACCCGGGATTCTCGCTTATTCTATTTTCTGATGTTAGCA
ATGTATAGTGGTCAAATAGGATTATTTTCTTCTCAAGATCTTTTACTTTTTTTCATCATG
TGGGAATTAGAATTAATTCCCGTTTATCTACTTTTATCCATGTGGGGGGGAAAGAAACGT
CTATATTCAGCTACAAAGTTTATTTTGTATACTGCAGGAAGCTCCGTTTTTTTATTAATG
GGAGCCTTGGGTATTGCTTTATATGGTTCCAATGAACCAACATTCAATTTTGAAACATCA
GCCAATCAACCATATCCTGCGGTCCTAGAAATATTGTTCTATATTGGATTTCTTATTGCT
TTTGCTGTCAAATCGCCGATTATACCCTTACATACATGGTTACCGGACACCCACGGAGAA
GCACATTACAGTACTTGTATGCTTTTAGCCGGAATCTTATTAAAAATGGGAGCGTATGGA
TTAGTTCGAATCAATATGGAATTGTTACCCCACGCCCATTCTCTATTTTCCCCTTGGTTA
ATAATAGTAGGTGCAATGCAAATAATCTATGCAGCTTCAACATCTTCTGGTCAGCGAAAT
TTAAAAAAAAGAATAGCCTATTCTTCTGTATCTCATATGGGTTTCATAATTATAGGAATT
TACTCTATAAGTGATATGGGACTCAATGGGTCCATTTTACAAATAATATCACATGGATTT
ATTGGCGCTGCACTTTTTTTCTTGGCCGGAACAAGTTATGATAGAATACGTCTTGTTTAT
CTTGACGAAATGGGTGGAATGGCTACTCCAATGCCAAAAATATTCACACTATTCAATATC
TTATCACTAGCTTCCCTTGCATTACCGGGCATGAGTGGTTTTGTTTCGGAATTGATAGTC
TTTTTTGGGATACTTACCACAGAAAAATATCTTTTAATGCCAAAAATAATAATTTCTTTT
GTAATGGCAGTTGGAATGCTATTAACTCCTCTTTATTTATTATCAATGTTACGTCAGATG
TTCTATGGATACAAGTTATTTAATGCCCTAAACTCTTATTGTTTTGATTCTGGGCCGCGG
GAATTATTTGTTTCCATTTCGATCCTTCTGCCTGTAATAAGTATTGGTATTTACCCGGAT
TTTATTTTCTCACTATCAGTTGAGAAAGTCGAAGCTATCATGTCGACCTACTTTTCTAGG
TAA

>G. gummi-gutta_petD
ATGGGAGTAACAAAAAAACCTGACTTGAATGATCCTGTTTTAAGAGCTAAATTGGCTAAG
GGCATGGGTCATAATTATTACGGAGAACCCGCATGGCCGAACGATCTTTTATATATTTTC
CCAGTAGTAATTCTCGGAACTATTGCATGTAATGTAGGATTAGCGGTTCTAGAACCATCA
ATGATTGGCGAACCCGCGGATCCATTTGCAACTCCTTTGGAAATATTGCCTGAATGGTAT
TTCTTTCCTGTATTTCAAATACTTCGTACAGTTCCAAATAAGTTATTGGGTGTTCTTTTA
ATGGTTTCAGTACCCGCAGGATTATTAACAGTACCTTTTTTGGAAAATGTTAATAAATTC
CAAAATCCATTTCGTCGTCCGGTCGCGACAACTGTCTTTTTGATTGGTACCGTAGTGGCC
CTTTGGTTTGGTATTGGAGCAACTTTGCCTATTGATAAATCCTTAACTTTAGGTCTTTTT
CAAATTGATTCAATTGTAAAATCAAATAGCACTACGTATGTATCTAGGGAATAG

>G. gummi-gutta_clpP
ATGCCTATTGGTGTCCCAAAAGTTCCTTTTCGAAATCCTGGAGAGGACGATTCCATTTGG
ATTGACGTATACAACCGACTTTATCGAGAAAGATTACTTTTTTTAGGTCAAGATGTTGAT
AGCGAAATCTCAAATCAACTTATTGGGCTTATGGTCTATCTCAGTATAGAGAGTGAGACC
AAAGATTTGTATTTGTTTATAAACTCTCCTGGCGGATGGGTAATACCTGGAATAGCGATT
TATGATACTATGCAATTTGTGCGACCAGATGTACAAACAGTATGCATGGGATTAGCCGCT
TCAATGGGATCTTTTATCCTGGCCGGGGGAAAAATTACCAAACGTCTAGCATTCCCTCAC
GCTAGGGTAATGATCCATCAACCTATTGCTGGTTTTTATGAGGCACAAATAGGAGAATTT
GTCCTGGAAGCGGAAGAGCTACTTAAATTGCGCGAAATCATCACAAGGGTGTATGCTCAA
AGAACGGGCAAACCTTTATGGGTTGTATCCGAAGACATGGAAAGGGATGTTTTTATGTCA
GCAACAGAAGCCCAAGCTCATGGACTTGTTGATCTTGTAGCAGTTACATAA

>G. mangostana var Thailand_cemA
ATGAGGCGGGTTCATTTAAAATTTTCATACGAAAAAATGAAAAAAAAAAAAGCCCTTATT
TCCCTTCTATATTTTACATCTATAATATTGTTGCCCTGGTGGATCTCTTTTTCGTTTAAT
AAAAGTGTGGAATCTTGGGTTATTAATTGGTGGAATACTAGTCAATCTGAAATTTTTTTA

AATGCTATTCAAGAAAAGAGTAGTTTAGAAAAATTCATAGAATTAGAGGAACTCTTACTC
TTGGACGAAATGATAAAGGAATGCCCGGAAACACATCTACAAAAGTTTATTATCGCAATC
CACAAAGAAACGATCAAATTGCTCAAAATGTACAATGAGGAGCGTATCTATACTATTTTG
CACTTCTCAACCAATATAATCTGTTTCATTATTCTAAGTGGTTATTCGATTCTAGGTAAT
GAAGAACTTTTTCTTCTTAATTCTTGGGTTCAAGAATTTCTATATAACCTAAGTGATACA
ATAAAAGCTTTTTTTATTCTTTTATTAACCGATTTATGTATAGGATTCCATTCACCCCAC
GGCTGGGAACTACTGATTGGTTCTGTCTACAAGGATTTTGGATTTACTTATAACGATCAA
ATTATATCTGGACTTGTTTCCACTTTTCCAGTAATTATCGATACAATTTTTAAATATTGG
ATCTTCCATTATTTAAATCGTGTATCTCCGTCACTTGTAGTGATTTATCATTCAATGAAT
GACTGA

>G. mangostana var Thailand_petD
ATGGGAGTAACAAAAAAACCTGACTTGAATGATCCTGTTTTAAGAGCTAAATTGGCTAAA
GGCATGGGTCATAATTATTACGGAGAACCTGCATGGCCGAACGATCTTTTATATATTTTC
CCAGTAGTAATTCTGGGGACTATTGCATGTAATGTAGGATTAGCGGTTCTAGAACCATCA
ATGATTGGCGAACCCGCGGATCCATTTGCAACTCCTTTGGAAATATTGCCTGAATGGTAT
TTCTTTCCTGTATTTCAAATACTTCGTACAGTTCCAAATAAGTTATTGGGTGTTCTTTTA
ATGGTTTCAGTACCCGCAGGATTATTAACAGTACCTTTTTTGGAAAATGTTAATAAATTC
CAAAATCCATTTCGTCGTCCGGTCGCGACAACTGTCTTTTTGATTGGTACCGTAGTGGCC
CTTTGGTTAGGTATTGGAGCAACTTTGCCTATTGATAAATCCTTAACTTTAGGTCTTTTT
CAAATTGATTCAATTGTAAAATAA

>G. mangostana var Thailand_clpP
ATGCCTATTGGTGTCCCAAAAGTTCCTTTTCGAAATCCTGGAGAGGACGATTCCATTTGG
ATTGACGTATACAACCGACTTTATCGAGAAAGATTACTTTTTTTAGGTCAAGATGTTGAT
AGCGAAATCTCAAATCAACTTATTGGGCTTATGGTCTATCTCAGTATAGAGAGTGAGACC
AAAGATTTGTATTTGTTTATAAACTCTCCTGGCGGATGGGTAATACCTGGAATAGCTATT
TATGATACTATGCAATTTGTGCGACCAGATGTACAAACAGTATGCATGGGATTAGCCGCT
TCAATGGGATCTTTTATCCTGGCCGGGGGAAAAATTACCAAACGTCTAGCATTCCCTCAC
GCTAGGGTAATGATCCATCAACCTATTGCTGGTTTTTATGAGGCACAAATAGGAGAATTT
GTCCTGGAAGCGGAAGAGCTACTTAAATTGCGCGAAATCATCACAAGGGTGTATGCTCAA
AGAACGGGCAAACCTTTATGGGTTGTATCCGAAGACATGGAAAGGGATGTTTTTATGTCA
GCAACAGAAGCCCAAGCTCATGGACTTGTTGATCTTGTAGCAGTTACATAA

>G. oblongifolia_petD
ATGGGAGTAACAAAAAAACCTGACTTGAATGATCCTGTTTTAAGAGCTAAATTGGCTAAA
GGCATGGGTCATAATTATTACGGAGAACCCGCATGGCCGAACGATCTTTTATATATTTTC
CCAGTAGTAATTCTCGGAACTATTGCATGTAATGTAGGATTAGCGGTTCTAGAACCATCA
ATGATTGGCGAACCCGCGGATCCATTTGCAACTCCTTTGGAAATATTGCCTGAATGGTAT
TTCTTTCCTGTATTTCAAATACTTCGTACAGTTCCAAATAAGTTATTGGGTGTTCTTTTA
ATGGTTTCAGTACCCGCAGGATTATTAACAGTACCTTTTTTGGAAAATGTTAATAAATTC
CAAAATCCATTTCGTCGTCCGGTGGCGACAACTGTCTTTTTGATTGGTACCGTAGCGGCC
CTTTGGTTAGGTATTGGAGCAACTTTGCCTATTGATAAATCCTTAACTTTAGGTCTTTTT
CAAATTGATTCAATTGGAAAATCAAATAGCACTAGGTATGTATCTAGGGAATAG

>G. oblongifolia_clpP
ATGCCTATTGGTGTCCCAAAAGTTCCTTTTCGAAATCCTGGAGAGGACGATTCCATTTGG
ATTGACGTATACAACCGACTTTATCGAGAAAGATTACTTTTTTTAGGTCAAGATGTTGAT
AGCGAAATCTCAAATCAACTTATTGGACTTATGGTCTATCTCAGTATAGAGAGTGAGACC
AAAGATTTGTATTTGTTTATAAACTCTCCTGGCGGATGGGTAATACCTGGAATAGCGATT

TATGATACTATGCAATTTGTGCGACCAGATGTACAAACAGTATGCATGGGATTAGCCGCT
TCAATGGGATCTTTTATCCTGGCCGGGGGAAAAATTACCAAACGTCTAGCATTCCCTCAC
GCTAGGGTAATGATCCATCAACCTATTGCTGGTTTTTATGAGGCACAAATAGGAGAATTT
GTCCTGGAAGCGGAAGAGCTACTTAAATTGCGCGAAATCATCACAAGGGTGTATGCTCAA
AGAACGGGCAAACCTTTATGGGTTGTATCCGAAGACATGGAAAGGGATGTTTTTATGTCA
GCAACAGAAGCCCAAGCTCATGGACTTGTTGATCTTGTAGCAGTTACATAA

>G. oblongifolia_rps16
ATGATAAAACTTCGTTTGAAGCGATGTGGTAGAAACCAACGAACCATTTATCGAATCGTT
GCAATTTATGTTCGATCCCGAGCGGGGGGGCGAGATCTTCAGAAAGTGGGTTTTTATGAT
CCGATAAAAAAAAATCGATTTCAATATTAA

>G. paucinervis_rps12
ATGCCAACTATTAAACAACTTATTAGAAACACAAGACAGCCAATCAAAAATGTCACAAAA
TCCCCCGCTCTTGTGGGCTGTCCTCAGCGACGAGGAACGTGTACTAGGGTGTATACTATC
ACCCCCAAAAAACCAAATTCTGCCTTACGTAAAGTAGCCAGAGTACGATTAACCTCTGGA
TTTGAAATCACTGCTTATATACCTGGTATTGGCCATAATTTACAAGAACATTCTGTAGTC
TTAGTAAGAGGGGGAAGGGTTAAGGATTTACCCGGTGTGAGATATCACATTGTTCGAGGA
ACCCTAGATGCTGTCGGAGTAAAGGATCGTCAACAAGGGCGTTCTAAATATGGAGTCAAA
AAGCCAAAATAA

>G. paucinervis_petD
ATGGATTCAACAAAAAAACCTGACTTGAATGATCCTGTTTTAAGAGCTAAATTGGCTAAA
GGCATGGGTCATAATTATTACGGAGAACCTGCATGGCCGAACGATCTTTTATATATTTTC
CCAGTAGTAATTCTCGGAACTATTGCATGTAATGTAGGATTAGCGGTTCTAGAACCATCA
ATGATTGGCGAACCCGCGGATCCATTTGCAACTCCTTTGGAAATATTGCCTGAATGGTAT
TTCTTTCCTGTATTTCAAATACTTCGTACAGTTCCAAATAAGTTATTGGGTGTTCTTTTA
ATGGTTTCAGTACCCGCAGGATTATTAACAGTACCTTTTTTGGAAAATGTTAATAAATTC
CAAAATCCATTTCGTCGTCCGGTCGCGACAACTGTCTTTTTGATTGGTACCGTGGTAGCC
CTTTGGTTAGGTATTGGAGCAACTTTGCCTATTGATAAATCCTTAACTTTGGGTCTTTTT
CAAATTGATTCAATTGTAAAATCAAATAGCACTACGTATGTATCTAGGGAATAG

>G. paucinervis_clpP
ATGCCTATTGGTGTCCCAAAAGTTCCTTTTCGAAATCCGGGAGAGGACGATTCCATTTGG
ATTGACGTATACAACCGACTTTATCGAGAAAGATTACTTTTTTTAGGTCAAGATGTTGAT
AGCGAAATCTCAAATCAACTTATTGGACTTATGGTCTATCTCAGTATAGAGAGTGAGACC
AAAGATTTGTATTTGTTTATAAACTCTCCTGGCGGATGGGTAATACCTGGAATAGCTATT
TATGATACTATGCAATTTGTGCGACCAGATGTACAAACAGTATGCATGGGATTAGCCGCT
TCAATGGGATCTTTTATCCTGGCCGGGGGAAAAATTACCAAACGTCTAGCATTCCCTCAC
GCTAGGGTAATGATCCATCAACCTATTGCTGGTTTTTATGAGGCACAAATAGGAGAATTT
GTCCTGGAAGCGGAAGAGCTACTTAAATTGCGCGAAATCATCACAAGGGTGTATGCTCAA
AGAACGGGCAAACCTTTATGGGTTGTATCCGAAGACATGGAAAGGGATGTTTTTATGTCA
GCAACAGAAGCCCAAGCTCATGGACTTGTTGATCTTGTAGCAGTTACATAA
>G. pedunculata_psbM
ATGGAAGTAAATATTCTCGCCTTTATTGCTACTGCACTCTTCATTCTAGTTCCTACTGCT
TTTTTGCTTATAATATACGTAAAAACTGTTAGTCAAAGCGATTAA

>G. pedunculata_ndhK
ATGAATTCCATTGAGTTTCCCCTACTTGATCGGACAACCCAAACTTCAGTTATTTCAACT
ACATCAAATGATCTTTCAAATTGGTCACGACTCTCCAGTTTATGGCCGCTTCTCTATGGT

ACCAGTTGTTGCTTCATTGAATTTGCTGCATTAATAGGCTCACGATTCGACTTTGATCGT
TATGGACTAGTACCAAGATCTAGTCCTAGACAGGCCGACCTTATTTTAACAGCTGGCACA
GTAACCATGAAAATGGCTCCTTCTTTAGTGAGATTATATGAACAAATGCCTGAACCAAAA
TATGTTATTGCTATGGGAGCCTGTACAATTACAGGAGGAATGTTCAGTACCGATTCTTAT
AGTACTGTTCGGGGAGTGGATAAGTTAATTCCTGTCGATGTCTATTTGCCAGGTTGTCCA
CCTAAACCGGAGGCCGTTATAGATGCTATAACAAAACTTCGTAAAAAACTATCTCGAGAA
ATTTATGACGATCGAATTCGGTCCCCACAGGGAAATCAGTGTTTTACTACCAATCATAAG
TTTCATATTGGATGCACTACTCATACCGGAAGTTATGATCAAGGATTGCTCTATCAACCG
CCGACTACTTCCAAAATTCCCCCTGAAACATTTTTCAAATACAAAAAGCCAGTCTCGTCC
TACGAATTAATAAATTAG

>G. pedunculata_ndhE
ATGATGCTCGAACATGTACTTGTTTTGAGTGCCTATTTATTTTCTATCGGTATCTATGGA
TTGATCACGAGTCGAAATATGGTTAGGGCCCTTATGTGCCTTGAACTTATTTTGAATGCT
GTTAATATCAATTTTGTAACATTTTCTGATTTTTTTGATAGTCGACAATTAAAAGGAAAT
ATTTTTTCCATTTTTGTTATAGCTATTGCAGCAGCTGAAGCGGCTATCGGGCTGGCTATT
GTTTCGTCAATTTATCGTAACAGAAAATCCATCCGTATCAATCAATCTAATTTGTTGAAT
AAGTAA

>G. pedunculata_cemA
ATGAGGCGGGTTCATTTAAAATTTTCATACGAAAAAATGAAAAAAAAAAAAAGCCCTTATT
TCCCTTCTATATTTTACATCTATAATATTGTTGCCCTGGTGGATCTCTTTTTCGTTTAAT
AAAAGTGTGGAATCTTGGGTTATTAATTGGTGGAATACTAGTCAATCTGAAATTTTTTTT
AATGCTATTCAAGAAAAGAGTAGTTTAGAAAAATTCATAGAATTAGAGGAACTCTTACTC
TTGGACGAAATGATAAAGGAATGCCCGGAAACACATCTACAAAAGTTTATTATCGCAATC
CACAAAGAAACGATCAAATTGCTCAAAATGTACAATGAGGAGCGTATCTATACTATTTTG
CACTTCTCAACCAATATAATCTGTTTCATTATTCTAAGCGGTTATTCGATTCTAGGTAAT
GAAGAACTTTTTCTTCTTAATTCTTGGGTTCAAGAATTTCTATATAACCTAAGTGATACA
ATAAAAGCTTTTTTTATTCTTTTATTAACCGATTTATGTATAGGATTCCATTCACCCCAC
GGCTGGGAACTACTGATTGGTTCTGTCTACAAGGATTTTGGATTTACTTATAACGATCAA
ATTATATCTGGACTTGTTTCCACTTTTCCAGTAATTATCGATACAATTTTAAAATATTGG
ATCTTCCATTATTTAAATCGTGTATCTCCGTCACTTGTAGTGATTTATCATTCAATGAAT
GACTGA

>G. pedunculata_ndhA
ATGAATATAACAGACATAATTGATATAATAGAAATTAATTCTTTTTCTAGATTGAGATTG
GAATCTTTAAACGAGATCTATGGAATTCTATGGATGCTTTTCCCTATTTTTATTCTGATA
TTGGGAATCACGATAGGTGTACTAGTAATTGTGTGGTTAGAAAGAGAAATATCTGCAGGG
GTGCAACAACGTATTGGACCTGAGTATGCCGGTCCTTTTGGAGTTCTTCAAGCGCTAGCG
GACGGGACAAAATTACTTTTTAAAGAGAATCTTTTTCCGTCTCGAGGAGATACTCGTTTA
TTCAGTCTTGGACCAGCCATAGCAGTCATATCAACTCTATTAAGCTATTCCGTAATTCCT
TTTAGCTATCACCTTGTTTTAGCTGATCTAACTATTGGTGTTTTTTTATGGATTGCCATT
TCAAGTATTGCCCCTATTGGCCTTCTTATGTCAGGGTATGGATCAAACAATAAATATTCC
TTTTTAGGTGGTCTACGAGCTGCTGCTCAATCAATTAGTTATGAAATACCATTAACTCTC
TGTGTATTATCCATATCTCTATTATCTAACAGTTCAAGTACAGTTGATATAGTTGAAGCA
CAATCAAAATATGGTTTTTGGGGGTGGAATTTTTGGCGTCAACCTATAGGGTTTATCATT
TTTTTAATTTCTTCCTTAGCGGAATGTGAGAGATTGCCCTTTGATTTACCAGAAGCAGAA
GAAGAATTAGTAGCAGGTTATCAAACCGAATATTCGGGTATTAAATTTGGTTTATTTTAT
GTTGCTTCCTATCTAAACTTATTAGTTTCGTCATTATTTGTAACAGTTCTTTACTTGGGT
GGTTGGAATATCTCTATTCCATTGCTTCCTGAATTTTTTGAGCTAACTAAAGTAAATGGA

GTCTGTGGAACAATAATGGGGATCTTTATTACATTAGTTAAAAGTTATTTGTTCTTGTTC
ATTTCTATCACAATAAGATGGACTTTACCTAGACTAAGAATGGATCAACTATTAAATCTT
GGATGGAAATTTCTTTTACCTATATCTCTTGGTAATTTAGTATTAACAACTTCTTTCCAA
TTACTTTCACTCTAA

>G. pedunculata_ndhD
ACGAATTCTTTTCCTTGGTTAACATTATTTGTAGTTTTCCCGATATCTGCGGGTTTTTTA
ATTTTCTTTTTACCTCATAGAGGTAATAAGGTAATTCGCTGGTATGCTATAAGTATTTCT
ATTTTAGAGCTCCTTTTAATGACTTATATATTTTCGTATTATTTCAAATTGGATGATCCA
TTAATACAATTAACAGAGAGTTCTAAATGGATCAATTTTTTGAATTTTTACTGGAGATTG
GGAATAGATGGGATCTCTTTAGGACCTATTTTTTTGACCGGATTTATCACTACTTTAGCT
ACTTTAGCGGCTCGGCCAGTTACCCGGGATTCTCGCTTATTCTATTTTCTGATGTTAGCA
ATGTATAGTGGTCAAATAGGATTATTTTCTTCTCAAGATCTTTTACTTTTTTTCATCATG
TGGGAATTAGAATTAATTCCCGTTTATCTACTTTTATCCATGTGGGGGGGAAAGAAACGT
CTATATTCAGCTACAAAGTTTATTTTGTATACTGCAGGAAGCTCCGTTTTTTTATTAATG
GGAGCCTTGGGTATTGCTTTATATGGTTCCAATGAACCAACATTCAATTTTGAAACATCA
GCCAATCAACCATATCCCGCGGTCCTAGAAATATTATTCTATATTGGATTTCTTGTTGCT
TTTGCTGTCAAATCGCCGATTATACCCTTACATACATGGTTACCGGACACCCACGGAGAA
GCACATTACAGTACTTGTATGCTTTTAGCCGGAATCTTATTAAAAATGGGAGCGTACGGA
TTAGTTCGAATCAATATGGAATTGTTACCCCACGCCCATTCTATATTTTCCCCTTGGTTA
ATAATAGTAGGTGCAATGCAAATAATCTATGCAGCTTCAACATCTTCTGGTCAGCGAAAT
TTAAAAAAAAGAATAGCCTATTCTTCTGTATCTCATATGGGTTTCATAATTATAGGAATT
TACTCTATAAGTGATATGGGACTCAATGGGGCCATTTTACAAATAATATCACATGGATTT
ATTGGCGCTGCACTTTTTTTCTTGGCCGGAACAAGTTATGATAGAATACGTCTTGTTTAT
CTTGACGAAATGGGCGGAATGGCTACTCCAATGCCAAAAATATTCACACTATTCAATATC
TTATCACTAGCTTCCCTTGCATTACCGGGCATGAGTGGTTTTTTTTCGGAATTGATAGTC
TTTTTGGGGATACTTACCACAGAAAAATATCTTTTAATGTCAAAAATAATAATTTCTTTT
GTAATGGCAGTTGGAATGCTATTAACTCCTCTTTATTTATTATCAATGTTACGTCAGATG
TTCTATGGATACAAGTTATTTAATGGCCTAAACTCTTATTGTTTTGATTCTGGGCCGCGG
GAATTATTTGTTTCCATTTCGATCCTTCTGCCTGTAATAAGTATTGGTATTTACCCGGAT
TTTATTTTCTCACTATCAGTTGAGAAAGTCGAAACTATCATGTTGACCTATTTTTCTAGG
TAA
