# Supplementary material for: Plastomes of Garcinia mangostana L. and Comparative Analysis with Other Garcinia Species
Source: Plants (Basel). 2023 Feb 17;12(4):930. doi: 10.3390/plants12040930 (PMC9966718; doi:10.3390/plants12040930)
Supplement: Supplementary file 1 [file plants-12-00930-s001.zip › Supplementary- Figures S1-S13.pdf]

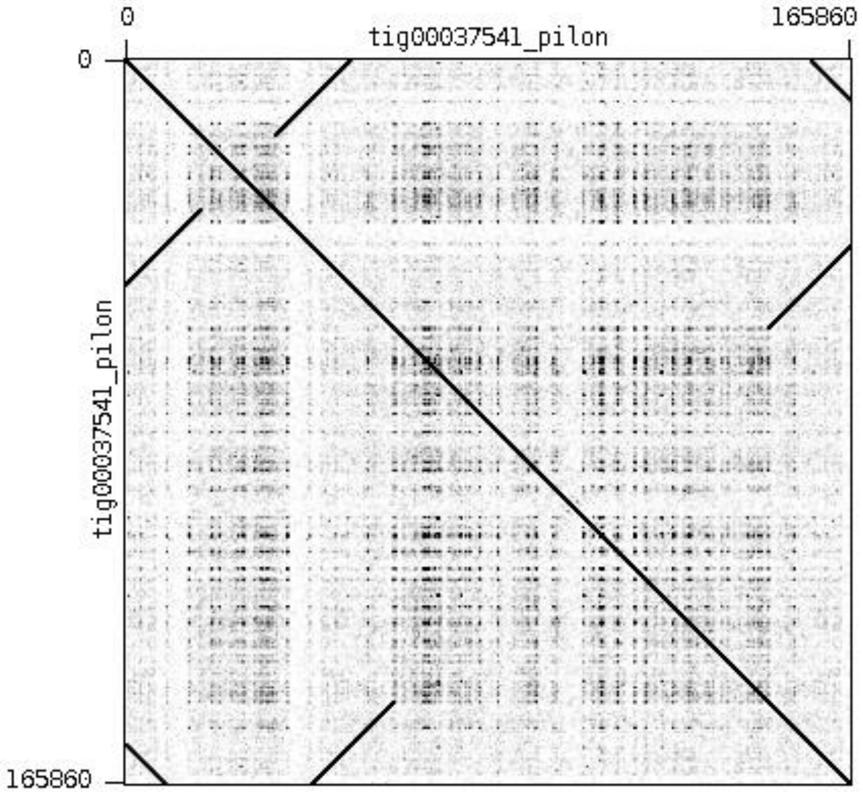

Figure S1. Dot plot analysis showing tig00037451_pilon as a circular contig.

a

b

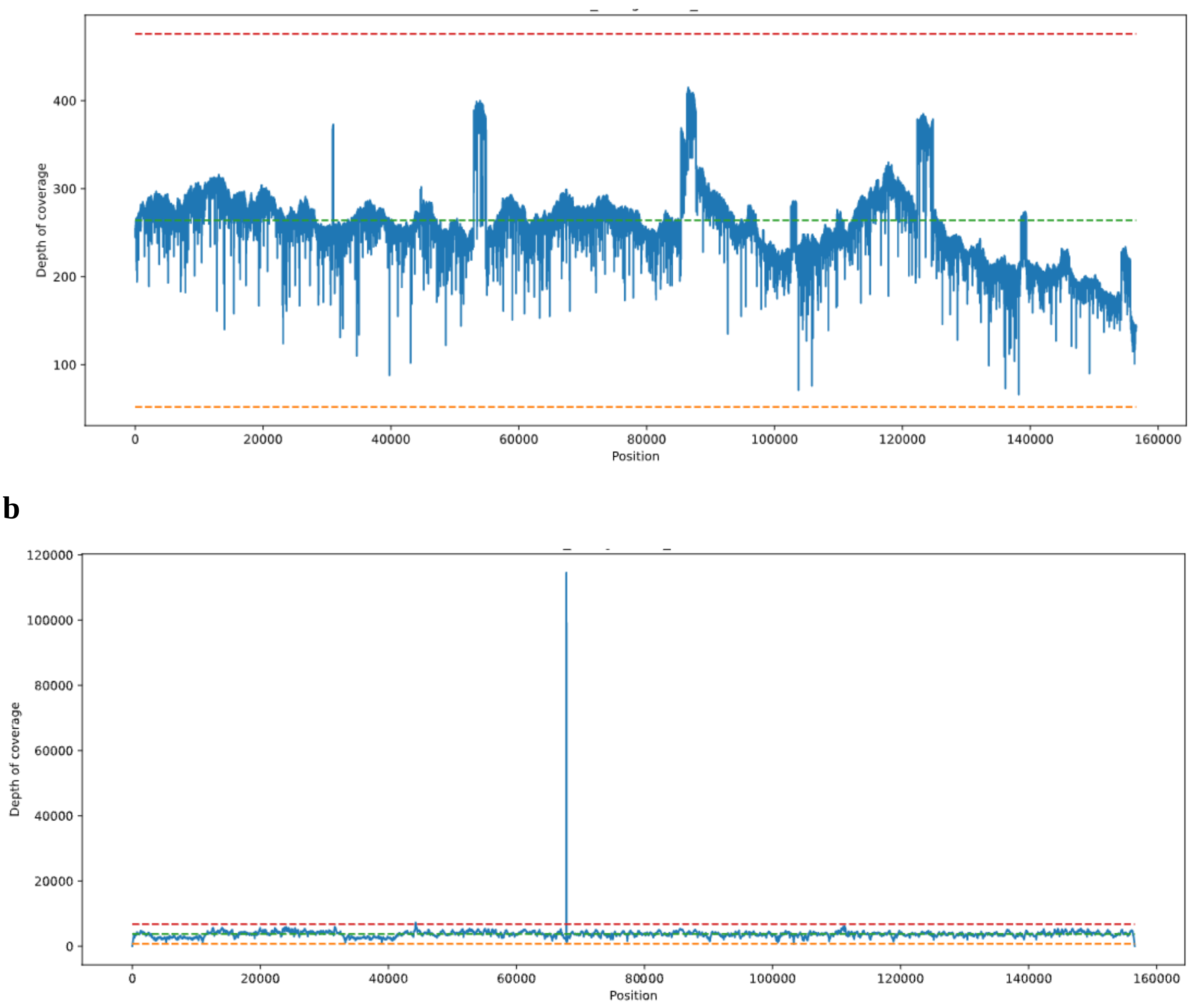

Figure S2. Read depth of (a) Mesta PacBio subreads and (b) Mesta Illumina clean reads mapped against the Mesta plastome (MZ823408). * Sharp peak in (b) is due to the TA-rich region.

a

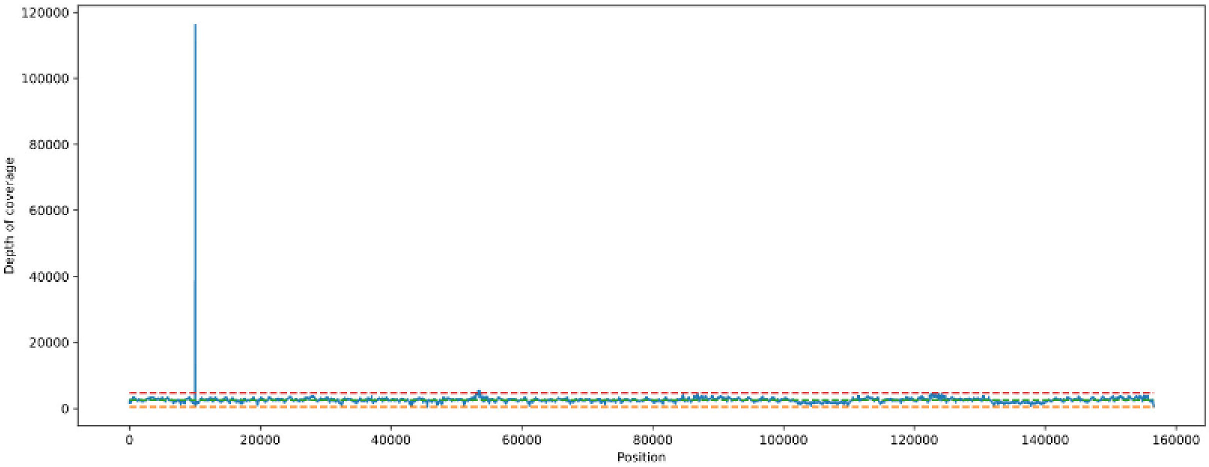
Depth of coverage
Position
0
20000
40000
60000
80000
100000
120000
140000
160000

b

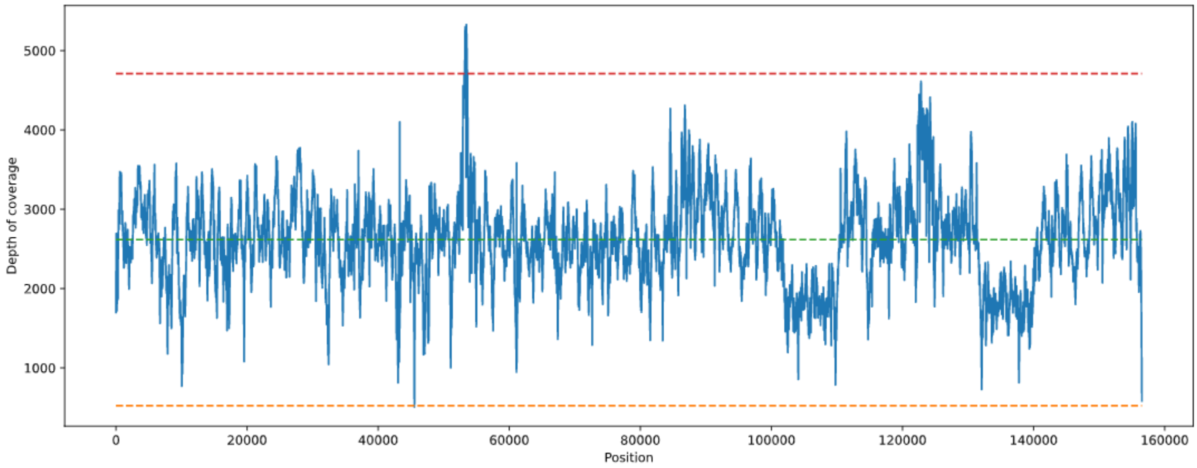
Depth of coverage
Position
1000
2000
3000
4000
5000
0
20000
40000
60000
80000
100000
120000
140000
160000

c

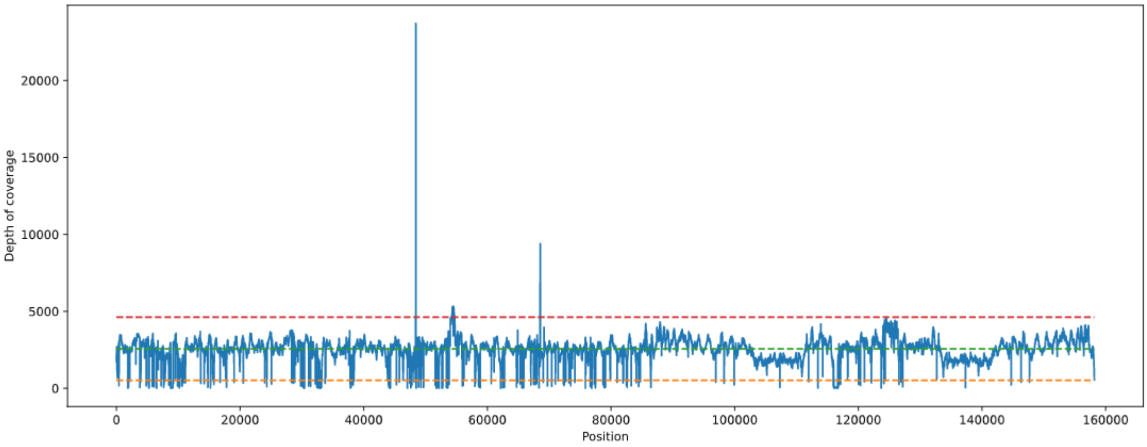
Depth of coverage
Position
0
5000
10000
15000
20000
0
20000
40000
60000
80000
100000
120000
140000
160000

d

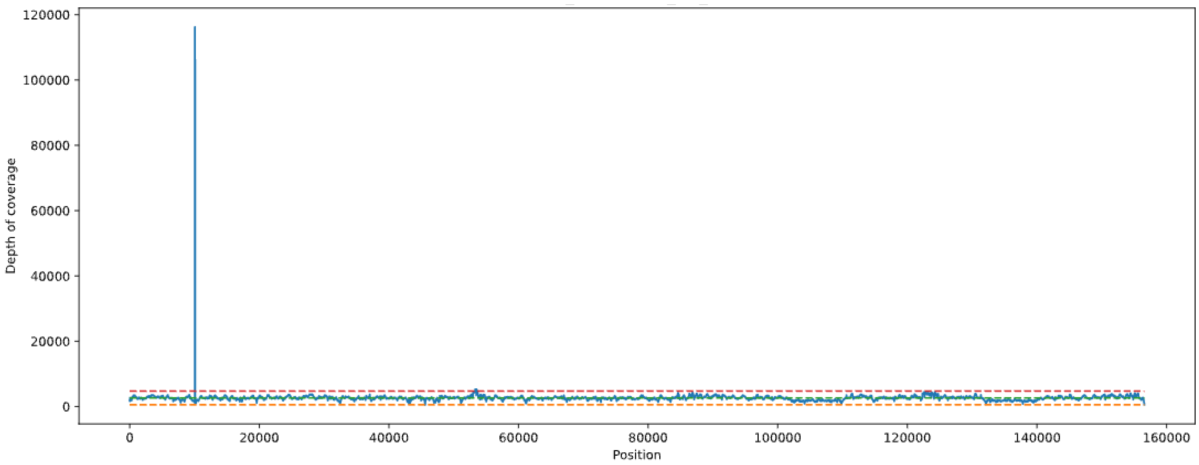

e

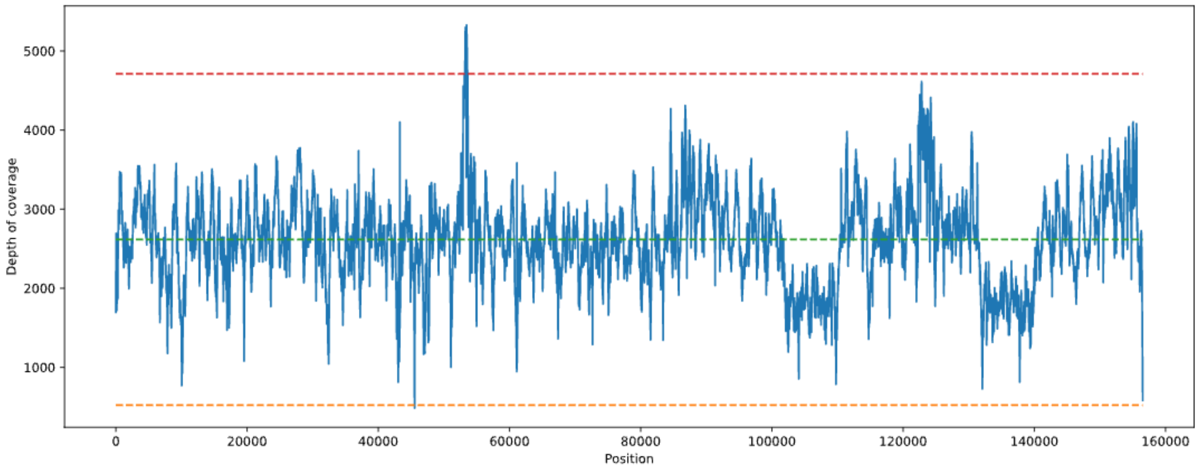

Figure S3. Read depth of Manggis Illumina clean reads mapped against plastomes of (a) Mesta variety, (b) Mesta*, (c) Thailand variety, (d) Manggis variety, and (e) Manggis*. *Edited to exclude the TA-rich region of 52 bp.

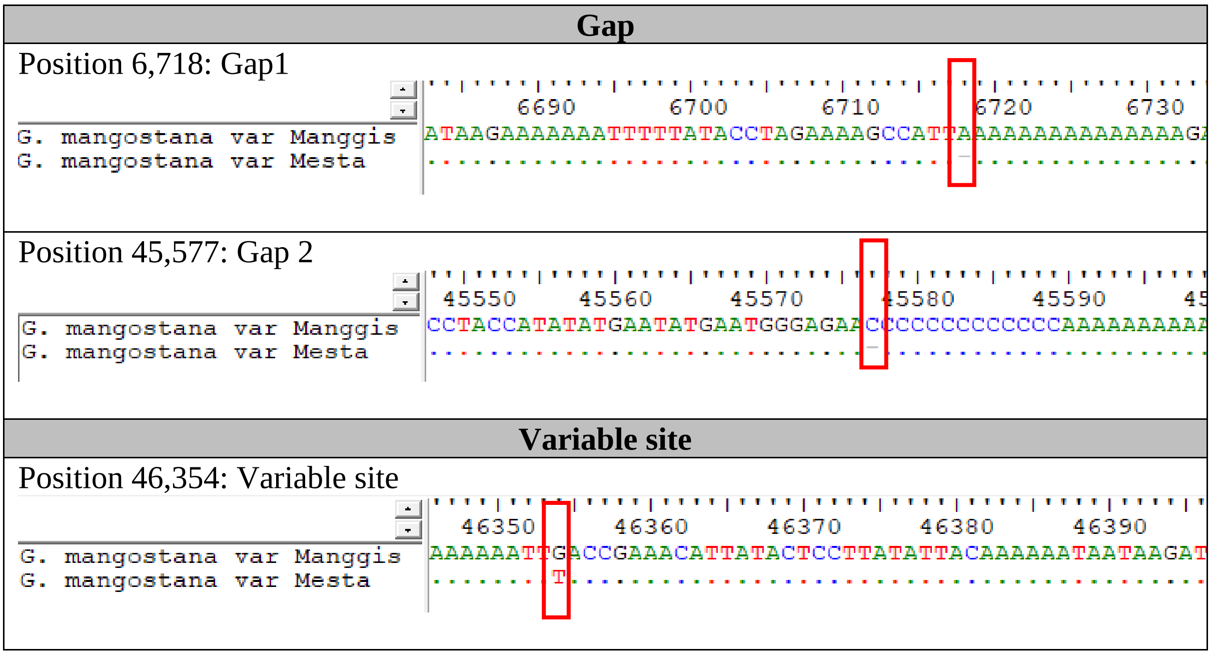

Figure S4. Sequence alignment result between plastomes of *G. mangostana* var. Manggis and var. Mesta showing the position of gaps and a variable site.

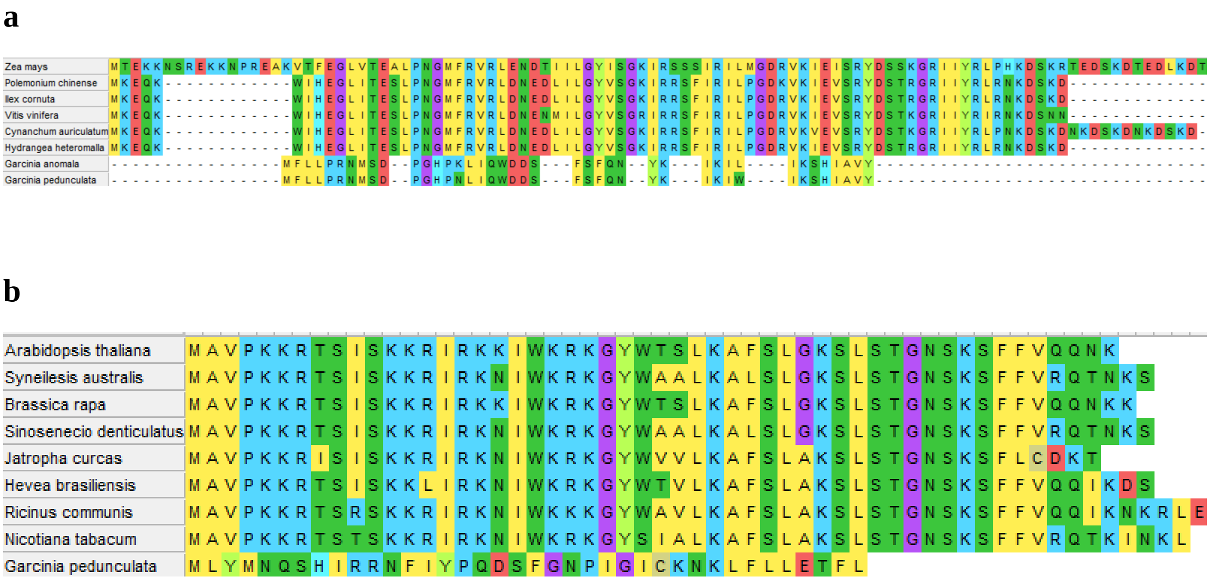

Figure S5. Multiple sequence alignment of (a) *infA* gene of *G. anomala* and *G. pedunculata*, (b) *rpl32* gene of *G. pedunculata* with other species.

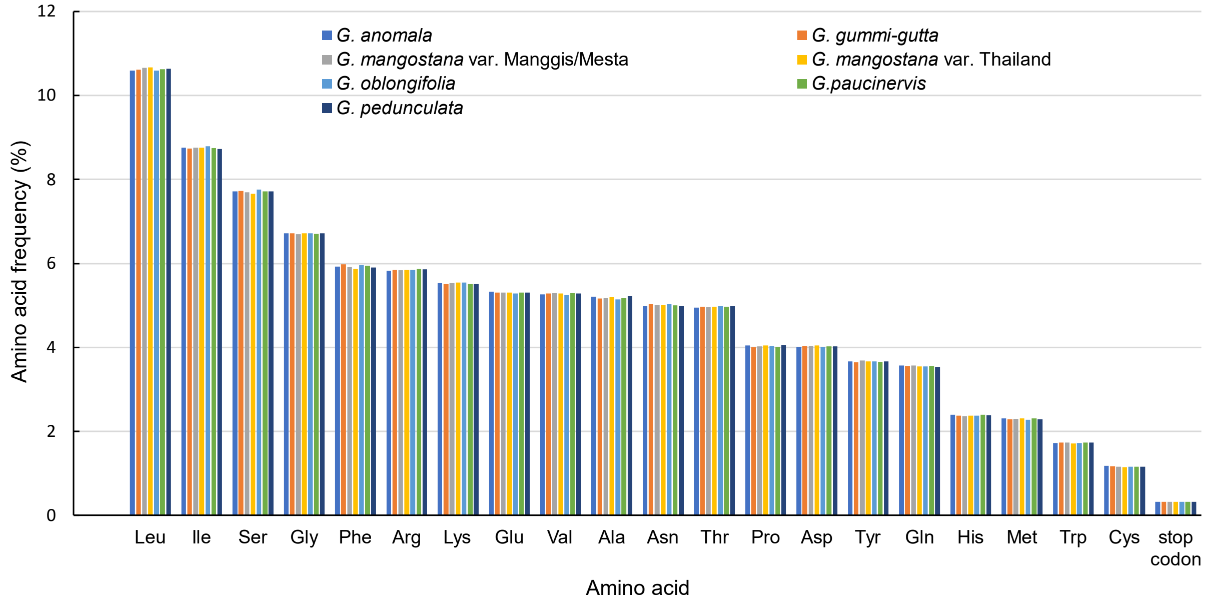

Figure S6. Comparison of amino acid frequency of six *Garcinia* species.

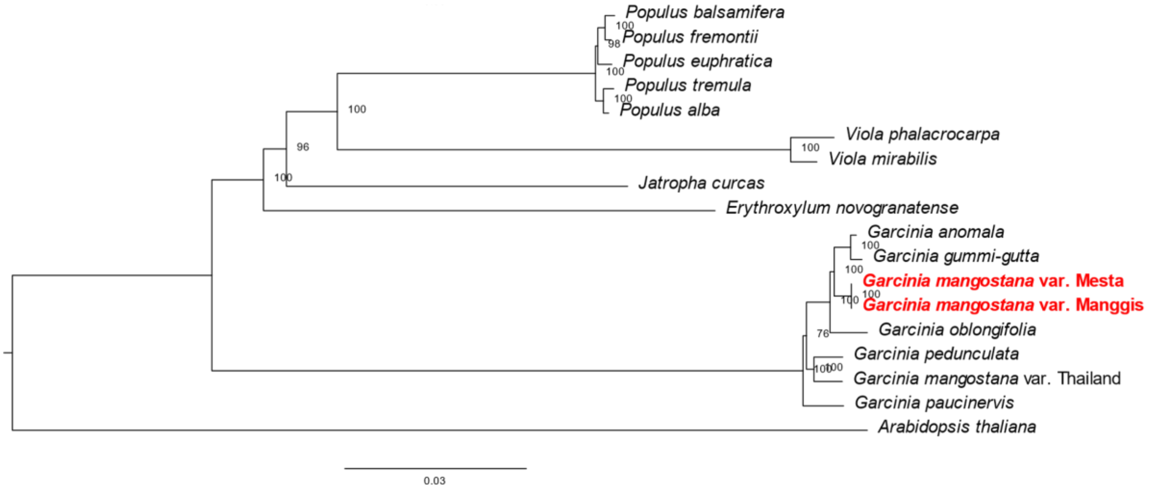

Figure S7. Phylogenetic tree (maximum likelihood) construction based on 16 species (three varieties from *G. mangostana*) whole plastome sequence.

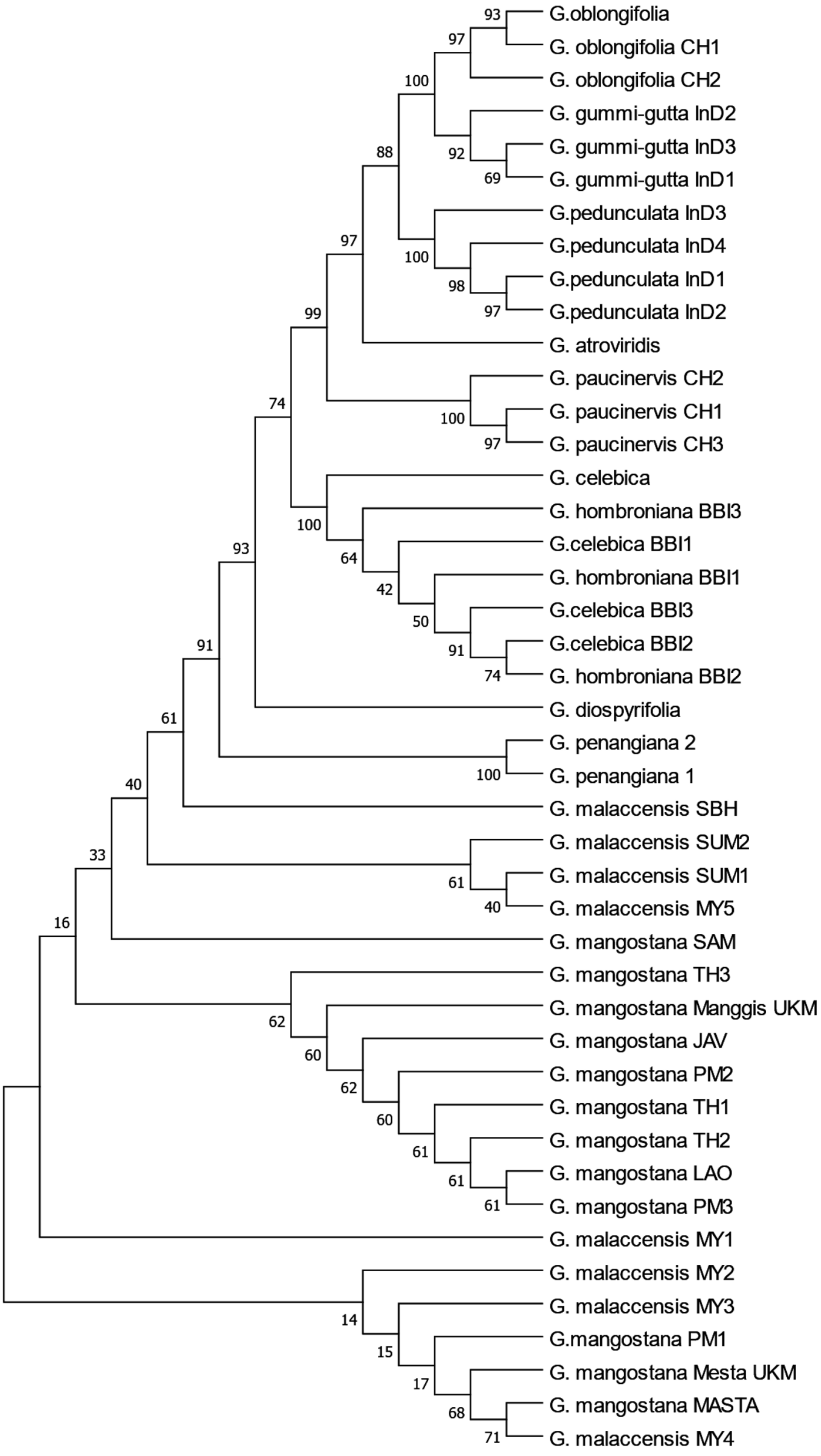

Figure S8. Phylogenetic tree inferred from the ITS gene of *Garcinia* species using Neighbor-Joining method with bootstrap replications set to 1000 (MEGA X Version 10.2.1). The evolutionary distances were computed using the Kimura 2-parameter method and are in the units of the number of base substitutions per site.

	26	115	200	249	444	477	490	527	616
G. mangostana LAO	CCG	CCC	TTC	TTA	ACG	CCC	GCT	GCA	AAA
G. mangostana JAV	CCG	CCC	TTC	TTA	ACG	CCC	GCT	GCA	AAA
G. mangostana TH1	CCG	CCC	TTC	TTA	ACG	CCC	GCT	GCA	AAA
G. mangostana TH2	CCG	CCC	TTC	TTA	ACG	CCC	GCT	GCA	AAA
G. mangostana PM2	CCG	CCC	TTC	TTA	ACG	CCC	GCT	GCA	AAA
G. mangostana PM3	CCG	CCC	TTC	TTA	ACG	CCC	GCT	GCA	AAA
G. mangostana TH3	CCG	CCC	TYC	TTA	ACG	CCC	GCT	GCA	AAA
G. mangostana SAM	CCG	CCC	TCC	-TA	ACG	CCC	GCT	GCA	ATA
G. mangostana MASTA	CCG	CCC	TCC	TTA	ACG	CCC	GCT	GTA	AAA
G. mangostana PM1	CCG	CCC	TCC	TTA	ACG	CCC	GCT	GCA	AAA
G. mangostana Manggis UKM	CCG	CCC	TYC	TTA	ACG	CCC	GCT	GCA	AAA
G. mangostana Mesta UKM	CCG	CCC	TCC	TTA	ASG	CMC	GCT	GYA	AAA
G. malaccensis MY1	CCG	CCC	TCC	TTA	ACG	CCC	GCT	GCA	AAA
G. malaccensis MY2	CCG	CCC	TCC	TTA	ACG	CCC	GCT	GCA	AAA
G. malaccensis MY3	CCG	CCC	TCC	TTA	ACG	CCC	GCT	GCA	AAA
G. malaccensis SUM2	CCG	CTC	TCC	TTA	AYG	CCC	GCT	GCA	AAA
G. malaccensis SUM1	CYG	CTC	TCC	TTA	ACG	CCC	GCT	GCA	AAA
G. malaccensis MY4	CCG	CCC	TCC	TTA	ACG	CCC	GCT	GTA	AAA
G. malaccensis MY5	CCG	CTC	TCC	TTA	ACG	CCC	GCT	GCA	AAA
G. malaccensis SBH	CCG	CCC	TCC	TTA	ACG	CCC	GTT	GCA	AAA

Figure S9. Comparison between *G. mangostana* L. (mangosteen) and *G. malaccensis* to identify the substitution sites and indels in internal transcribed spacer (ITS) sequences.

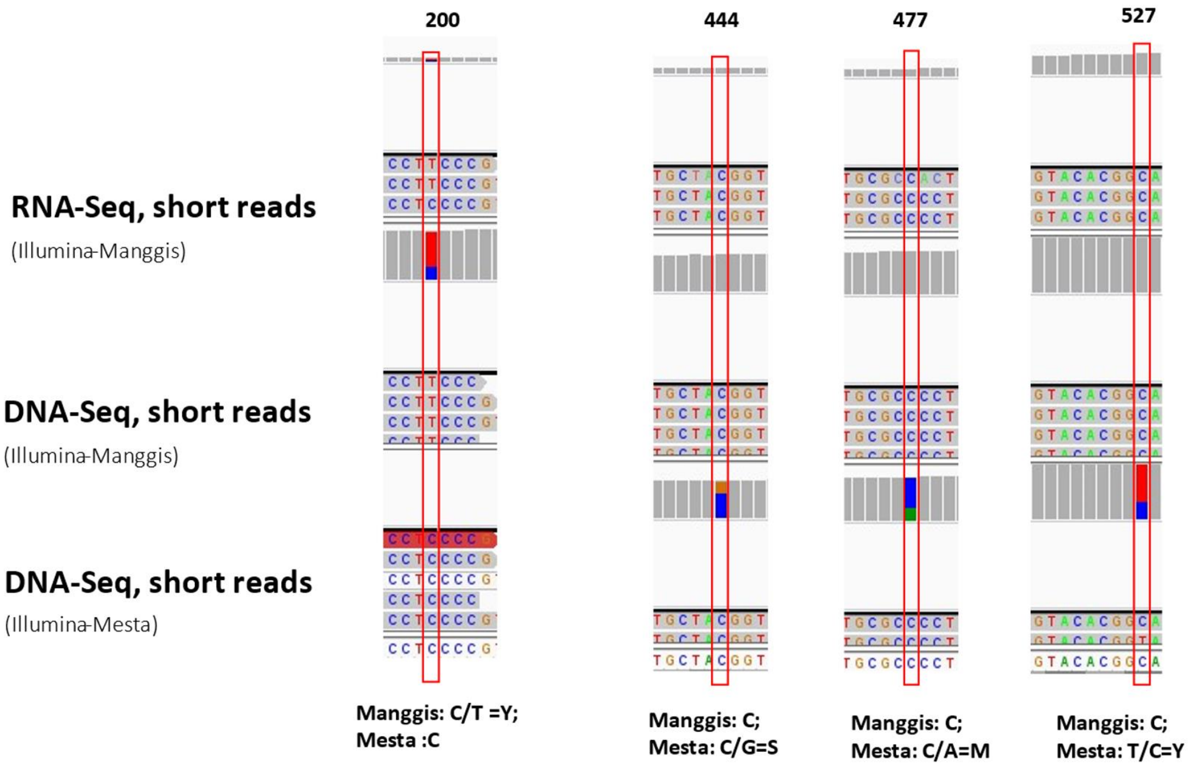

Figure S10. Positions of ITS gene with heterozygosity detected at ITS gene of Manggis and Mesta varieties as visualized using IGV.

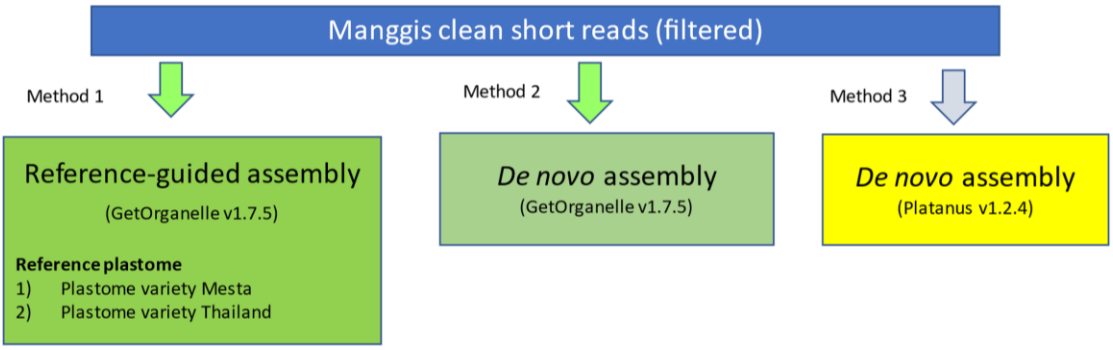

Figure S11. Different methods used in Manggis variety plastome assembly.

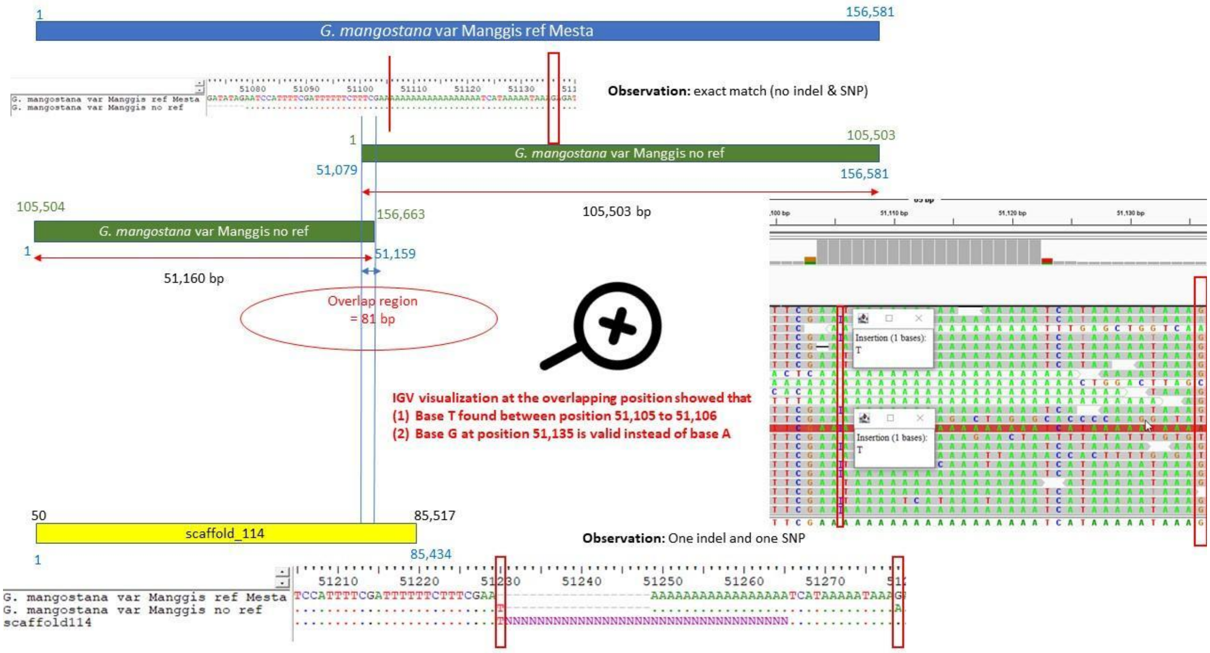

Figure S12. ClustalW alignment and IGV visualization to confirm the indel and SNP detected in Supplementary Table S12. ClustalW alignment was performed to detect the position of the one indel and one SNP while IGV tool was used to confirm the existence of indel and SNP. IGV visualization confirmed that base T existed between position of 51,105 and 51,106 while position 51,136 confirmed was base G. Hence, base T was added between position of 51,105 and 51,106 of *G. mangostana* var Manggis ref Mesta.

Original ORF (*cemA*)

```
G. anomala                          --------------------MKKKN-LISLLYFISIIILLPWWISFSFNK
G. gummi-gutta                      MEIVNCIKSTNEAGSFKIRKNEKK-NLISLLYFISIIILLPWWISFSFNK
G. mangostana var Manggis/Mesta     MRRV--------HLK--YEKMKKN--LISLLYFISIIILLPWWISFSFNK
G. mangostana var Thailand          --------------------MKKKKALISLLYFTSIIILLPWWISFSFNK
G. oblongifolia                     MEIVNCIKSTNEAGSFKIRKNEKKKNLISLLYFISIIILLPWWISFSFNK
G. paucinervis                      MRRV--------HLKFSYEKMKKKKALISLLYFTSIIILLPWWISFSFNK
G. pedunculata                      --------------------MKKKKALISLLYFTSIIILLPWWISFSFNK
```

ORF adjustment at 5’ end (*cemA*)
Species with CDS adjusted: *G. anomala*, *G. mangostana* var Thailand, *G. pedunculata*

```
G. anomala                          MRRV--------HLK--YEKMKKKN-LISLLYFISIIILLPWWISFSFNK
G. gummi-gutta                      MEIVNCIKSTNEAGSFKIRKNEKK-NLISLLYFISIIILLPWWISFSFNK
G. mangostana var Manggis/Mesta     MRRV--------HLK--YEKMKKN--LISLLYFISIIILLPWWISFSFNK
G. mangostana var Thailand          MRRV--------HLKFSYEKMKKKKALISLLYFTSIIILLPWWISFSFNK
G. oblongifolia                     MEIVNCIKSTNEAGSFKIRKNEKKKNLISLLYFISIIILLPWWISFSFNK
G. paucinervis                      MRRV--------HLKFSYEKMKKKKALISLLYFTSIIILLPWWISFSFNK
G. pedunculata                      MRRV--------HLKFSYEKMKKKKALISLLYFTSIIILLPWWISFSFNK
```

Original ORF (*petD*)

```
G. anomala                          MGVTKKPDLNDPVLRAKLAKGMGHNYYGEPAWPNDLLYIFPVVILGTIA
G. gummi-gutta                      MGA-KKPDLNDPVLRAKLAKGMGHNYYGEPAWPNDLLYIFPVVILGTIA
G. mangostana var Manggis/Mesta     MGVTKKPDLNDPVLRAKLAKGMGHNYYGEPAWPNDLLYIFPVVILGTIA
G. mangostana var Thailand          MGA-KKPDLNDPVLRAKLAKGMGHNYYGEPAWPNDLLYIFPVVILGTIA
G. oblongifolia                     MGA-KKPDLNDPVLRAKLAKGMGHNYYGEPAWPNDLLYIFPVVILGTIA
G. paucinervis                      MDS-KKPDLNDPVLRAKLAKGMGHNYYGEPAWPNDLLYIFPVVILGTIA
G. pedunculata                      MGVTKKPDLNDPVLRAKLAKGMGHNYYGEPAWPNDLLYIFPVVILGTIA
```

ORF adjustment at splicing site (*petD*)
Species with CDS adjusted:
G. gummi-gutta, *G. mangostana* var Thailand, *G. oblongifolia*, *G. paucinervis*

```
G. anomala                          MGVTKKPDLNDPVLRAKLAKGMGHNYYGEPAWPNDLLYIFPVVILGTIA
G. gummi-gutta                      MGVTKKPDLNDPVLRAKLAKGMGHNYYGEPAWPNDLLYIFPVVILGTIA
G. mangostana var Manggis/Mesta     MGVTKKPDLNDPVLRAKLAKGMGHNYYGEPAWPNDLLYIFPVVILGTIA
G. mangostana var Thailand          MGVTKKPDLNDPVLRAKLAKGMGHNYYGEPAWPNDLLYIFPVVILGTIA
G. oblongifolia                     MGVTKKPDLNDPVLRAKLAKGMGHNYYGEPAWPNDLLYIFPVVILGTIA
G. paucinervis                      MDSTKKPDLNDPVLRAKLAKGMGHNYYGEPAWPNDLLYIFPVVILGTIA
G. pedunculata                      MGVTKKPDLNDPVLRAKLAKGMGHNYYGEPAWPNDLLYIFPVVILGTIA
```

Original ORF (*rps16*)

```
G. anomala                          MIKLRLKRCGRNQRTIYRIVAIDVRSRGEGRDLQKVGFYDPIKNKIYFN
G. gummi-gutta                      MIKLRLKRFGRNQRTIYRIVAIDVRSRGEGRDLQKVGFYDPIKNKIYFN
G. mangostana var Manggis/Mesta     MIKLRLKRCGRNQRTIYRIVAIDVRSRGEGRDLQKVGFYDPIKDKIYFN
G. mangostana var Thailand          MIKLRLKRCGRKQRTIYRIVAIDVRSRGEGRDLQKVGFYDPIKNKIYSN
G. oblongifolia                     MIKLRLKRCGRNQHEPF-IESLQF-------------MFDPERGGEIFR
G. paucinervis                      MIKLRLKRCGRKQRTIYRIVAIDVRSRGEGRDLQKVGFYDPIKKKIYFN
G. pedunculata                      MIKLRLKRCGRKRRTIYRIVAIDVRSRGEGRDL----------------
```

ORF adjustment at splicing site (*rps16*)
Species with CDS adjusted: *G. oblongifolia*

```
G. anomala                          MIKLRLKRCGRNQRTIYRIVAIDVRSRGEGRDLQKVGFYDPIKNKIYFN
G. gummi-gutta                      MIKLRLKRFGRNQRTIYRIVAIDVRSRGEGRDLQKVGFYDPIKNKIYFN
G. mangostana var Manggis/Mesta     MIKLRLKRCGRNQRTIYRIVAIDVRSRGEGRDLQKVGFYDPIKDKIYFN
G. mangostana var Thailand          MIKLRLKRCGRKQRTIYRIVAIDVRSRGEGRDLQKVGFYDPIKNKIYSN
G. oblongifolia                     MIKLRLKRCGRNQRTIYRIVAIYVRSRAGGRDLQKVGFYDPIKKNRFCY
G. paucinervis                      MIKLRLKRCGRKQRTIYRIVAIDVRSRGEGRDLQKVGFYDPIKKKIYFN
G. pedunculata                      MIKLRLKRCGRKRRTIYRIVAIDVRSRGEGRDL----------------
```

Figure S13. Example of ORF alignment before and after adjustment.
