# Supplementary material for: Plastomes of Garcinia mangostana L. and Comparative Analysis with Other Garcinia Species
Source: Plants (Basel). 2023 Feb 17;12(4):930. doi: 10.3390/plants12040930 (PMC9966718; doi:10.3390/plants12040930)
Supplement: Supplementary file 1 [file plants-12-00930-s001.zip › Supplementary -Tables S1-S14.pdf]

Table S1. Summary statistics of the polished assembled *G. mangostana* var. Mesta genome.

Features	*G. mangostana* var. Mesta
**Unfiltered subreads**	
Total number	1,040,386
Total bases (bp)	21,364,043,967
**Corrected and Trimmed subreads**	
Total number	208,217
Total bases (bp)	914,713,746
**Assembled polished data**	
Total length (bp)	58,761,678
Number of contigs	7,616
Largest contig size (bp)	395,099
N50 (bp)	10,212

Table S2. Summary statistics of the (a) Mesta PacBio subread and (b) Mesta Illumina clean read depth coverage mapped against the Mesta plastome (MZ823408).

Sequence data	Ref. Length	Mapped Reads	Breadth	% Coverage	Min. Depth	Max. Depth	Avg. Depth
PacBio	156,580	6,779	156,580	100	66	415	265
Illumina	156,580	5,987,076	156,580	100	18	114,565	3,751

Table S3. Summary statistics of Manggis Illumina clean data read depth coverage against plastomes of Thailand, Mesta, and Manggis varieties.

Variety	Length (bp)	No. mapped reads	Breadth	% Coverage	Min. Depth	Max. Depth	Avg. Depth
Thailand KX822787	158,179	4,177,616	158,126	99.97	0	23,706	2,566
Mesta	156,580	4,252,841	156,580	100	506	116,240	2,638
Mesta*	156,528	4,140,697	156,528	100	506	5,328	2,617
Manggis	156,582	4,252,794	156,582	100	484	116,240	2,636
Manggis*	156,530	4,140,650	156,530	100	484	5,328	2,617

*Edited to exclude the TA-rich region of 52 bp.

Table S4. Summary of the polymorphism site analysis.

Features	Polymorphism site between plastomes of Manggis and Mesta varieties
Number of sites	156,582
Sites with alignment gaps or missing data	2
Invariable (monomorphic) sites	156,579
Variable (polymorphic) sites	1

Table S5. Genes with intron(s) in the plastomes of *Garcinia* species. Gene *rps12** is a trans-spliced gene with 5'end located at the LSC regions while the duplicated 3' ends located at the IR regions.

Species	Gene	Location	Exon I (bp)	Intron I (bp)	Exon II (bp)	Intron II (bp)	Exon III (bp)
G. anomala	*rps16*	LSC	40	905	179		
	atpF	LSC	145	761	398		
	rpoC1	LSC	432	761	1632		
	ycf3	LSC	126	717	387		
	rps12	LSC	114		232	538	26
	clpP	LSC	71	753	292	613	228
	petB	LSC	6	852	642		
	petD	LSC	8	820	526		
	rpl16	LSC	9	1172	399		
	rpl2	IR	400	661	434		
	ndhB	IR	777	699	756		
	ndhA	SSC	561	1134	534		
	trnK-UUU	LSC	37	2564	35		
	trnG-UCC	LSC	23	703	48		
	trnL-UAA	LSC	35	644	50		
	trnV-UAC	LSC	39	610	35		
	trnI-GAU	IR	37	937	35		
	trnA-UGC	IR	38	803	35		
G. gummi-gutta	*rps16*	LSC	40	910	179		
	atpF	LSC	145	760	398		
	rpoC1	LSC	432	769	1632		
	ycf3	LSC	126	718	387		
	rps12	LSC	114		232	538	26
	clpP	LSC	71	748	292	613	228
	petB	LSC	6	851	642		
	petD	LSC	8	812	526		
	rpl16	LSC	9	1152	399		
	rpl2	IR	400	661	434		
	ndhB	IR	777	699	756		
	ndhA	SSC	562	1136	533		
	trnK-UUU	LSC	37	2589	35		
	trnG-UCC	LSC	23	703	48		
	trnL-UAA	LSC	35	619	50		
	trnV-UAC	LSC	39	606	35		
	trnI-GAU	IR	37	944	35		
	trnA-UGC	IR	38	804	35		

Table S5 (continue). Genes with intron(s) in the plastomes of *Garcinia* species. Gene *rps12** is a trans-spliced gene with 5'end located at the LSC regions while the duplicated 3' ends located at the IR regions.

Species	Gene	Location	Exon I (bp)	Intron I (bp)	Exon II (bp)	Intron II (bp)	Exon III (bp)
G. mangostana var. Manggis/ Mesta	*rps16*	LSC	40	908	179		
	atpF	LSC	145	751	398		
	rpoC1	LSC	432	755	1635		
	ycf3	LSC	126	722	387		
	rps12	LSC	114		232	538	26
	clpP	LSC	71	755	292	612	228
	petB	LSC	6	838	642		
	petD	LSC	8	798	526		
	rpl16	LSC	9	1217	399		
	rpl2	IR	400	661	434		
	ndhB	IR	777	697	756		
	ndhA	SSC	562	1157	533		
	trnK-UUU	LSC	37	2543	35		
	trnG-UCC	LSC	23	706	48		
	trnL-UAA	LSC	35	624	50		
	trnV-UAC	LSC	39	607	35		
	trnI-GAU	IR	37	945	35		
	trnA-UGC	IR	38	803	35		
G. mangostana var. Thailand	*rps16*	LSC	40	923	179		
	atpF	LSC	145	743	398		
	rpoC1	LSC	432	753	1632		
	ycf3	LSC	126	725	387		
	rps12	LSC	114		232	538	26
	clpP	LSC	71	736	292	637	228
	petB	LSC	6	829	642		
	petD	LSC	8	813	496		
	rpl16	LSC	9	1188	399		
	rpl2	IR	400	661	434		
	ndhB	IR	777	698	756		
	ndhA	SSC	562	1157	533		
	trnK-UUU	LSC	37	2560	35		
	trnG-UCC	LSC	23	696	48		
	trnL-UAA	LSC	35	658	50		
	trnV-UAC	LSC	39	596	35		
	trnI-GAU	IR	37	944	35		
	trnA-UGC	IR	38	795	35		
	trnI-GAU	IR	37	941	35		
	trnA-UGC	IR	38	804	35		

Table S5 (continue). Genes with intron(s) in the plastomes of *Garcinia* species. Gene *rps12** is a trans-spliced gene with 5'end located at the LSC regions while the duplicated 3' ends located at the IR regions.

Species	Gene	Location	Exon I (bp)	Intron I (bp)	Exon II (bp)	Intron II (bp)	Exon III (bp)
G. oblongifolia	*rps16*	LSC	40	908	110		
	atpF	LSC	145	761	398		
	rpoC1	LSC	432	758	1632		
	ycf3	LSC	126	722	387		
	rps12	LSC	114		232	538	26
	clpP	LSC	71	751	292	629	228
	petB	LSC	6	828	642		
	petD	LSC	8	798	526		
	rpl16	LSC	9	1200	399		
	rpl2	IR	400	669	434		
	ndhB	IR	777	700	756		
	ndhA	SSC	562	1086	533		
	trnK-UUU	LSC	37	2585	35		
	trnG-UCC	LSC	23	690	48		
	trnL-UAA	LSC	35	638	50		
	trnV-UAC	LSC	39	601	35		
	trnI-GAU	IR	37	944	35		
	trnA-UGC	IR	38	801	35		
G. paucinervis	*rps16*	LSC	40	773	224		
	atpF	LSC	145	748	398		
	rpoC1	LSC	432	753	1632		
	ycf3	LSC	126	729	387		
	rps12	LSC	114		232	538	26
	clpP	LSC	71	745	292	641	228
	petB	LSC	6	841	642		
	petD	LSC	8	22	526		
	rpl16	LSC	9	43	399		
	rpl2	IR	400	669	434		
	ndhB	IR	777	699	756		
	ndhA	SSC	561	1148	534		
	trnK-UUU	LSC	37	2552	35		
	trnG-UCC	LSC	23	717	48		
	trnL-UAA	LSC	35	640	50		
	trnV-UAC	LSC	39	597	35		
	trnI-GAU	IR	37	943	35		
	trnA-UGC	IR	38	801	35		

Table S5 (continue). Genes with intron(s) in the plastomes of *Garcinia* species. Gene *rps12** is a trans-spliced gene with 5'end located at the LSC regions while the duplicated 3' ends located at the IR regions.

Species	Gene	Location	Exon I (bp)	Intron I (bp)	Exon II (bp)	Intron II (bp)	Exon III (bp)
G. pedunculata	*rps16*	LSC	40	924	62		
	atpF	LSC	145	744	398		
	rpoC1	LSC	432	765	1632		
	ycf3	LSC	126	722	387		
	rps12	LSC	114		232	538	26
	clpP	LSC	71	756	292	648	228
	petB	LSC	6	804	642		
	petD	LSC	8	807	526		
	rpl16	LSC	9	1185	399		
	rpl2	IR	400	661	434		
	ndhB	IR	777	701	756		
	ndhA	SSC	561	1140	534		
	trnK-UUU	LSC	37	2559	35		
	trnG-UCC	LSC	23	694	48		
	trnL-UAA	LSC	35	637	50		
	trnV-UAC	LSC	39	597	35		
	trnI-GAU	IR	37	941	35		
	trnA-UGC	IR	38	804	35		

Table S6. Relative Synonymous Codon Usage (RSCU) in plastomes of different *Garcinia* species.

Codon	Amino acid	*G. anomala*		*G. gummi-gutta*		*G. mangostana*						*G. oblongifolia*		*G. paucinervis*		*G. pedunculata*	
						**Manggis**		**Mesta**		**Thailand**							
		**Count**	**RSCU**	**Count**	**RSCU**	**Count**	**RSCU**	**Count**	**RSCU**	**Count**	**RSCU**	**Count**	**RSCU**	**Count**	**RSCU**	**Count**	**RSCU**
GCU(A)	Ala	632	1.85	627	1.85	630	1.85	630	1.85	633	1.86	628	1.86	629	1.85	633	1.85
GCC(A)	Ala	221	0.65	221	0.65	221	0.65	221	0.65	220	0.65	223	0.66	220	0.65	223	0.65
GCA(A)	Ala	372	1.09	367	1.08	373	1.1	373	1.1	375	1.1	365	1.08	369	1.09	377	1.1
GCG(A)	Ala	143	0.42	141	0.42	135	0.4	135	0.4	133	0.39	134	0.4	139	0.41	133	0.39
CGU(R)	Arg	316	1.24	320	1.25	322	1.26	322	1.26	318	1.24	321	1.25	323	1.26	313	1.22
CGC(R)	Arg	95	0.37	94	0.37	94	0.37	94	0.37	101	0.4	97	0.38	92	0.36	107	0.42
CGA(R)	Arg	378	1.48	380	1.48	379	1.48	379	1.48	371	1.45	380	1.48	380	1.48	371	1.45
CGG(R)	Arg	116	0.46	113	0.44	113	0.44	113	0.44	114	0.45	111	0.43	113	0.44	118	0.46
AGA(R)	Arg	468	1.84	475	1.85	470	1.84	470	1.84	476	1.86	473	1.85	477	1.86	468	1.83
AGG(R)	Arg	156	0.61	155	0.61	156	0.61	156	0.61	154	0.6	155	0.61	155	0.6	159	0.62
AAU(N)	Asn	1029	1.57	1044	1.58	1035	1.57	1035	1.57	1034	1.57	1042	1.58	1032	1.57	1031	1.58
AAC(N)	Asn	279	0.43	279	0.42	281	0.43	281	0.43	281	0.43	279	0.42	279	0.43	277	0.42
GAU(D)	Asp	843	1.6	850	1.6	849	1.6	849	1.6	851	1.6	843	1.6	845	1.6	841	1.6
GAC(D)	Asp	209	0.4	210	0.4	209	0.4	209	0.4	210	0.4	211	0.4	211	0.4	212	0.4
UGU(C)	Cys	236	1.52	236	1.55	237	1.56	237	1.56	232	1.54	237	1.56	235	1.55	234	1.55
UGC(C)	Cys	74	0.48	69	0.45	67	0.44	67	0.44	69	0.46	67	0.44	69	0.45	68	0.45
CAA(Q)	Gln	744	1.59	742	1.59	743	1.59	743	1.59	734	1.58	741	1.59	741	1.59	734	1.59
CAG(Q)	Gln	193	0.41	191	0.41	193	0.41	193	0.41	196	0.42	191	0.41	192	0.41	192	0.41
GAA(E)	Glu	1075	1.54	1072	1.54	1074	1.54	1074	1.54	1071	1.54	1068	1.54	1073	1.54	1076	1.55
GAG(E)	Glu	323	0.46	322	0.46	318	0.46	318	0.46	320	0.46	321	0.46	320	0.46	315	0.45
GGU(G)	Gly	543	1.23	553	1.25	551	1.25	551	1.25	545	1.24	548	1.24	552	1.25	545	1.24
GGC(G)	Gly	207	0.47	196	0.44	194	0.44	194	0.44	204	0.46	200	0.45	199	0.45	203	0.46
GGA(G)	Gly	714	1.62	709	1.61	714	1.62	714	1.62	715	1.62	718	1.63	708	1.61	717	1.63
GGG(G)	Gly	298	0.68	305	0.69	299	0.68	299	0.68	297	0.67	298	0.68	301	0.68	296	0.67
CAU(H)	His	482	1.54	476	1.53	475	1.53	475	1.53	476	1.53	479	1.54	480	1.53	480	1.54

CAC(H)	His	145	0.46	147	0.47	146	0.47	146	0.47	145	0.47	145	0.46	148	0.47	145	0.46
AUU(I)	Ile	1152	1.5	1148	1.5	1153	1.5	1153	1.5	1154	1.51	1155	1.5	1151	1.5	1140	1.5
AUC(I)	Ile	415	0.54	419	0.55	422	0.55	422	0.55	416	0.54	423	0.55	423	0.55	420	0.55
AUA(I)	Ile	732	0.96	728	0.95	724	0.94	724	0.94	727	0.95	733	0.95	723	0.94	727	0.95
UUA(L)	Leu	925	1.99	923	1.99	929	1.99	929	1.99	927	1.99	912	1.97	924	1.99	918	1.98
UUG(L)	Leu	547	1.18	543	1.17	543	1.16	543	1.16	551	1.18	546	1.18	542	1.17	547	1.18
CUU(L)	Leu	617	1.33	618	1.33	617	1.32	617	1.32	617	1.32	619	1.33	616	1.33	616	1.33
CUC(L)	Leu	167	0.36	172	0.37	174	0.37	174	0.37	169	0.36	171	0.37	174	0.37	173	0.37
CUA(L)	Leu	359	0.77	361	0.78	365	0.78	365	0.78	362	0.78	368	0.79	362	0.78	363	0.78
CUG(L)	Leu	167	0.36	172	0.37	170	0.36	170	0.36	171	0.37	167	0.36	171	0.37	169	0.36
AAA(K)	Lys	1112	1.53	1102	1.52	1110	1.53	1110	1.53	1105	1.52	1108	1.52	1100	1.52	1104	1.53
AAG(K)	Lys	339	0.47	345	0.48	344	0.47	344	0.47	347	0.48	347	0.48	345	0.48	340	0.47
AUG(M)	Met	606	1	600	1	603	1	603	1	606	1	598	1	604	1	599	1
UUU(F)	Phe	1045	1.34	1060	1.35	1047	1.35	1047	1.35	1040	1.35	1051	1.34	1051	1.35	1045	1.35
UUC(F)	Phe	511	0.66	510	0.65	507	0.65	507	0.65	500	0.65	514	0.66	510	0.65	500	0.65
CCU(P)	Pro	437	1.65	436	1.66	437	1.66	437	1.66	443	1.67	436	1.65	437	1.66	437	1.65
CCC(P)	Pro	189	0.71	186	0.71	185	0.7	185	0.7	186	0.7	189	0.71	186	0.71	189	0.71
CCA(P)	Pro	293	1.1	289	1.1	293	1.11	293	1.11	285	1.08	288	1.09	290	1.1	295	1.11
CCG(P)	Pro	143	0.54	141	0.54	141	0.53	141	0.53	145	0.55	146	0.55	140	0.53	140	0.53
UCU(S)	Ser	553	1.64	562	1.66	556	1.65	556	1.65	552	1.65	555	1.63	559	1.66	563	1.67
UCC(S)	Ser	329	0.97	323	0.95	324	0.96	324	0.96	326	0.97	323	0.95	324	0.96	325	0.96
UCA(S)	Ser	409	1.21	406	1.2	404	1.2	404	1.2	403	1.2	413	1.22	405	1.2	402	1.19
UCG(S)	Ser	201	0.59	200	0.59	202	0.6	202	0.6	200	0.6	208	0.61	200	0.59	199	0.59
AGU(S)	Ser	388	1.15	394	1.16	386	1.15	386	1.15	386	1.15	392	1.15	393	1.17	387	1.15
AGC(S)	Ser	147	0.44	145	0.43	148	0.44	148	0.44	141	0.42	147	0.43	143	0.42	145	0.43
ACU(T)	Thr	543	1.67	551	1.69	549	1.69	549	1.69	545	1.67	549	1.68	551	1.69	544	1.67
ACC(T)	Thr	209	0.64	201	0.62	202	0.62	202	0.62	207	0.64	204	0.62	200	0.61	207	0.63
ACA(T)	Thr	416	1.28	418	1.28	417	1.28	417	1.28	413	1.27	417	1.28	420	1.29	418	1.28
ACG(T)	Thr	129	0.4	134	0.41	133	0.41	133	0.41	137	0.42	137	0.42	132	0.41	135	0.41
UGG(W)	Trp	451	1	454	1	454	1	454	1	449	1	453	1	454	1	453	1

UAU(Y)	Tyr	802	1.67	797	1.66	807	1.67	807	1.67	801	1.67	802	1.67	798	1.66	799	1.67
UAC(Y)	Tyr	160	0.33	161	0.34	160	0.33	160	0.33	161	0.33	160	0.33	161	0.34	160	0.33
GUU(V)	Val	514	1.49	521	1.5	516	1.48	516	1.48	514	1.48	516	1.5	523	1.51	517	1.49
GUC(V)	Val	172	0.5	169	0.49	170	0.49	170	0.49	169	0.49	164	0.48	168	0.48	168	0.49
GUA(V)	Val	501	1.45	510	1.47	511	1.47	511	1.47	508	1.47	510	1.48	508	1.46	509	1.47
GUG(V)	Val	195	0.56	189	0.54	193	0.56	193	0.56	195	0.56	189	0.55	191	0.55	191	0.55
UAA(*)	stop codon	44	1.59	44	1.59	44	1.59	44	1.59	44	1.59	43	1.55	44	1.59	43	1.55
UAG(*)	stop codon	23	0.83	22	0.8	22	0.8	22	0.8	22	0.8	22	0.8	22	0.8	24	0.87
UGA(*)	stop codon	16	0.58	17	0.61	17	0.61	17	0.61	17	0.61	18	0.65	17	0.61	16	0.58
**Total number of codon usage**		26,249		26,265		26,257		26,257		26,216		26,268		26,244		26,195	

Table S7. SSRs identified on the plastome of Mesta variety.

No.	Type	SSR	Size	Start	End	Region
1	p1	(A)13	13	286	298	LSC
2	p1	(A)10	10	2149	2158	LSC
3	p1	(A)14	14	6718	6731	LSC
4	c	(T)13atttgaaaatgaaaagattttagattggataagtttaaagacggattttttgtctaccttactttactttaaaactttaaatc(T)12	108	7111	7218	LSC
5	p1	(A)17	17	7860	7876	LSC
6	p1	(A)10	10	8126	8135	LSC
7	p1	(A)10	10	9141	9150	LSC
8	p1	(T)12	12	9282	9293	LSC
9	p2	(TA)12	24	10060	10083	LSC
10	p1	(T)10	10	10404	10413	LSC
11	p1	(A)11	11	12428	12438	LSC
12	p1	(A)12	12	14994	15005	LSC
13	p2	(AT)7	14	15335	15348	LSC
14	p1	(T)12	12	16420	16431	LSC
15	p1	(T)14	14	17324	17337	LSC
16	p1	(T)16	16	19585	19600	LSC (*rpoC2*)
17	p1	(A)10	10	22222	22231	LSC (*rpoC1*)
18	c	(A)12gagctactccttactcaagttcccaacgaagaccaagcaccaattcattcttctttgttttgtcca(T)11	89	23673	23761	LSC
19	p1	(T)10	10	27327	27336	LSC (*rpoB*)
20	c	(T)11ctcattttttgccc(T)10	35	30537	30571	LSC
21	c	(A)10ttaacagtctatttagagtttaatgatttagaatatttgaatttctaatgatatatattatatacaattagtatacaattagaaattcaaaaattga(T)13	120	32391	32510	LSC
22	p1	(T)13	13	36387	36399	LSC
23	p1	(G)10	10	43078	43087	LSC
24	c	(T)12cgttttctttaatttctttaaaataaaatatatatatttctttataagagataataagagaaaagaacgaacc(TA)9	103	43248	43350	LSC
25	c	(C)12(A)12	24	45576	45599	LSC
26	p2	(TA)7	14	46972	46985	LSC
27	p1	(A)16	16	47165	47180	LSC
28	p1	(A)12	12	47384	47395	LSC
29	c	(TA)6attaatataattaatattttatttttattttaattttaggaatatgaaaaaaattgtcttgaatcaatcccaagttcaagaatcagaattg(A)14	118	47650	47767	LSC
30	p1	(T)10	10	50153	50162	LSC

31	p1	(T)12	12	50955	50966	LSC
32	p1	(A)17	17	51105	51121	LSC
33	p1	(T)10	10	51759	51768	LSC
34	c	(A)10tgttcgatatcaagtttctcggttaattcaataagaaatcgaagttagtactcgattttgttggtaccatacaacgaattgaattcaa(T)11ct attttgcaaatcagttagttgaacttgaaaattcattgattgaaatag(A)15	174	54853	55026	LSC
35	p1	(T)10	10	55177	55186	LSC
36	p1	(A)10	10	57049	57058	LSC
37	c	(T)12aactta(T)10	28	57579	57606	LSC
38	p1	(T)10	10	60008	60017	LSC
39	p2	(AT)8	16	61164	61179	LSC
40	c	(T)13acaaaaatttggaattctatctagtgttctagtgttgataagaagactatttgattttatctcttctttcg(T)13ctctaaatctaaattggggg gtgattatgtcactattctattgtcagatttaactgttatcgaatgtattaatag(T)10	182	63643	63824	LSC
41	p1	(T)11	11	66702	66712	LSC
42	p1	(A)15	15	67466	67480	LSC
43	p3	(TAT)5	15	69306	69320	LSC
44	c	(T)10c(A)11	22	71353	71374	LSC
45	p1	(A)10	10	72038	72047	LSC
46	p3	(TAA)5	15	72182	72196	LSC
47	p1	(T)11	11	74912	74922	LSC
48	p1	(A)11	11	76296	76306	LSC
49	p2	(AT)6	12	80709	80720	LSC
50	p1	(T)10	10	80921	80930	LSC
51	p1	(A)11	11	82170	82180	LSC
52	p1	(A)10	10	83263	83272	LSC
53	p1	(A)11	11	83984	83994	LSC
54	p1	(T)10	10	85316	85325	LSC (*rps19*)
55	p1	(T)11	11	85638	85648	IRA
56	p1	(A)13	13	91398	91410	IRA (*ycf2*)
57	p1	(T)10	10	98588	98597	IRA
58	p1	(T)11	11	104626	104636	IRA
59	p1	(A)12	12	109667	109678	IRA
60	p1	(T)13	13	109823	109835	IRA
61	p1	(A)14	14	114948	114961	SSC
62	p1	(T)13	13	116651	116663	SSC
63	p1	(A)12	12	121020	121031	SSC

64	c	(T)13cgttcctcttcttcgttcggaaaaaaagggggcttagcctaaattcgaataaataaagcaaaggattctttcgttcctgatagtca(T)10	109	122071	122179	SSC
65	p1	(T)13	13	126256	126268	SSC (*ycf1*)
66	p1	(T)10	10	126962	126971	SSC (*ycf1*)
67	p1	(T)12	12	127472	127483	SSC (*ycf1*)
68	p1	(T)10	10	128012	128021	SSC (*ycf1*)
69	p1	(A)13	13	132129	132141	IRB
70	p1	(T)12	12	132286	132297	IRB
71	p1	(A)11	11	137328	137338	IRB
72	p1	(A)10	10	143367	143376	IRB
73	p1	(T)13	13	150554	150566	IRB (*ycf2*)
74	p1	(A)11	11	156316	156326	IRB
**Gene name in parentheses indicates SSR that is found within the CDS of the respective gene.**						

p1 = mononucleotide; p2 = dinucleotide; p3 = trinucleotide; c = compound microsatellite

Table S8. SSRs identified on the plastome of Manggis variety.

No.	Type	SSR	Size	Start	End	Region
1	p1	(A)13	13	286	298	LSC
2	p1	(A)10	10	2149	2158	LSC
3	p1	(A)15	15	6718	6732	LSC
4	c	(T)13atttgaaaatgaaaagattttagattggataagtttaaagacggatttttgtctaccttactttactttaaaactttaaatc(T)12	108	7112	7219	LSC
5	p1	(A)17	17	7861	7877	LSC
6	p1	(A)10	10	8127	8136	LSC
7	p1	(A)10	10	9142	9151	LSC
8	p1	(T)12	12	9283	9294	LSC
9	p2	(TA)12	24	10061	10084	LSC
10	p1	(T)10	10	10405	10414	LSC
11	p1	(A)11	11	12429	12439	LSC
12	p1	(A)12	12	14995	15006	LSC
13	p2	(AT)7	14	15336	15349	LSC
14	p1	(T)12	12	16421	16432	LSC
15	p1	(T)14	14	17325	17338	LSC
16	p1	(T)16	16	19586	19601	LSC (*rpoC2*)
17	p1	(A)10	10	22223	22232	LSC (*rpoC1*)
18	c	(A)12gagctactccttactcaagttcccaacgaagaccaagcaccaattcattcttctttgttttgtcca(T)11	89	23674	23762	LSC
19	p1	(T)10	10	27328	27337	LSC (*rpoB*)
20	c	(T)11ctcattttttgccc(T)10	35	30538	30572	LSC
21	c	(A)10ttaacagtctatttagagtttaatgatttagaatatttgaatttctaatgatatatattatatacaattagtatacaattagaaattcaaaaattga (T)13	120	32392	32511	LSC
22	p1	(T)13	13	36388	36400	LSC
23	p1	(G)10	10	43079	43088	LSC
24	c	(T)12cgttttctttaatttctttaaaataaaatatatatatttctttataagagataataagagaaaagaacgaacc(TA)9	103	43249	43351	LSC
25	c	(C)13(A)12	25	45577	45601	LSC
26	p2	(TA)7	14	46974	46987	LSC
27	p1	(A)16	16	47167	47182	LSC
28	p1	(A)12	12	47386	47397	LSC
29	c	(TA)6attaatataattaatattttatttttattttaattttaggaatatgaaaaaaattgtcttgaatcaatcccaagttcaagaatcagaattg(A)1 4	118	47652	47769	LSC
30	p1	(T)10	10	50155	50164	LSC
31	p1	(T)12	12	50957	50968	LSC

32	p1	(A)17	17	51107	51123	LSC
33	p1	(T)10	10	51761	51770	LSC
34	c	(A)10tgttcgatatcaagtttctcggttaattcaataagaaatcgaagttagtactcgattttgttggtaccatacaacgaattgaattcaa(T)11 ctattttgcaaatcagttagttgaacttgaaaattcattgattgaaatag(A)15	174	54855	55028	LSC
35	p1	(T)10	10	55179	55188	LSC
36	p1	(A)10	10	57051	57060	LSC
37	c	(T)12aactta(T)10	28	57581	57608	LSC
38	p1	(T)10	10	60010	60019	LSC
39	p2	(AT)8	16	61166	61181	LSC
40	c	(T)13acaaaaatttggaattctatctagtgttctagtgttgataagaagactatttgattttatctcttctttcg(T)13ctctaaatctaaattgggg ggtgattatgtcactattctattgtcagatttaactgttatcgaatgtattaatag(T)10	182	63645	63826	LSC
41	p1	(T)11	11	66704	66714	LSC
42	p1	(A)15	15	67468	67482	LSC
43	p3	(TAT)5	15	69308	69322	LSC
44	c	(T)10c(A)11	22	71355	71376	LSC
45	p1	(A)10	10	72040	72049	LSC
46	p3	(TAA)5	15	72184	72198	LSC
47	p1	(T)11	11	74914	74924	LSC
48	p1	(A)11	11	76298	76308	LSC
49	p2	(AT)6	12	80711	80722	LSC
50	p1	(T)10	10	80923	80932	LSC
51	p1	(A)11	11	82172	82182	LSC
52	p1	(A)10	10	83265	83274	LSC
53	p1	(A)11	11	83986	83996	LSC
54	p1	(T)10	10	85318	85327	LSC (*rps19*)
55	p1	(T)11	11	85640	85650	IR
56	p1	(A)13	13	91400	91412	IR (*ycf2*)
57	p1	(T)10	10	98590	98599	IR
58	p1	(T)11	11	104628	104638	IR
59	p1	(A)12	12	109669	109680	IR
60	p1	(T)13	13	109825	109837	IR
61	p1	(A)14	14	114950	114963	SSC
62	p1	(T)13	13	116653	116665	SSC
63	p1	(A)12	12	121022	121033	SSC
64	c	(T)13cgttcctcttcttcgttcggaaaaaaagggggcttagcctaaattcgaataaataaagcaaaggattctttcgttcctgatagtca(T)10	109	122073	122181	SSC

65	p1	(T)13	13	126258	126270	SSC (*ycf1*)
66	p1	(T)10	10	126964	126973	SSC (*ycf1*)
67	p1	(T)12	12	127474	127485	SSC (*ycf1*)
68	p1	(T)10	10	128014	128023	SSC (*ycf1*)
69	p1	(A)13	13	132131	132143	IR
70	p1	(T)12	12	132288	132299	IR
71	p1	(A)11	11	137330	137340	IR
72	p1	(A)10	10	143369	143378	IR
73	p1	(T)13	13	150556	150568	IR (*ycf2*)
74	p1	(A)11	11	156318	156328	IR
**Gene name in parentheses indicates SSR that is found within the CDS of the respective gene.**						

p1 = mononucleotide; p2 = dinucleotide; p3 = trinucleotide; c = compound microsatellite

Table S9. GenBank accession numbers of the species used the study.

Species		Order	Family	GenBank accession no
Arabidopsis thaliana		Brassicales	Brassicaceae	NC_000932
Erythroxylum novogranatense		Malpighiales	Erythroxylaceae	NC_030601
Garcinia anomala		Malpighiales	Clusiaceae	MW582313
Garcinia gummi-gutta		Malpighiales	Clusiaceae	NC_047250
Garcinia mangostana	Manggis	Malpighiales	Clusiaceae	OK572535
	Mesta	Malpighiales	Clusiaceae	MZ823408
	Thailand	Malpighiales	Clusiaceae	KX822787
Garcinia oblongifolia		Malpighiales	Clusiaceae	NC_050384
Garcinia paucinervis		Malpighiales	Clusiaceae	MT501656
Garcinia pedunculata		Malpighiales	Clusiaceae	NC_048983
Jatropha curcas		Malpighiales	Euphorbiaceae	NC_012224
Populus alba		Malpighiales	Salicaceae	NC_008235
Populus balsamifera		Malpighiales	Salicaceae	NC_024735
Populus euphratica		Malpighiales	Salicaceae	NC_024747
Populus fremontii		Malpighiales	Salicaceae	NC_024734
Populus tremula		Malpighiales	Salicaceae	NC_027425
Viola mirabilis		Malpighiales	Violaceae	NC_041582
Viola phalacrocarpa		Malpighiales	Violaceae	NC_041583

Table S10. List of protein-coding genes used to construct phylogenomics tree.

Protein coding-genes used to construct phylogenetic tree					
psbA	*psbD*	*accD*	*rps12*	*rpl22*	*ndhA*
matK	*psbC*	*psaI*	*clpP*	*rps19*	*ndhH*
psbK	*psbZ*	*cemA*	*psbT*	*rpl2*	*rps15*
psbI	*rps14*	*petA*	*psbN*	*rpl23*	*ycf1*
atpA	*psaB*	*psbJ*	*psbH*	*ycf2*	
atpF	*psaA*	*psbL*	*petB*	*ndhB*	
atpH	*ycf3*	*psbF*	*petD*	*rps7*	
atpI	*rps4*	*psbE*	*rpoA*	*ndhF*	
rps2	*ndhJ*	*petL*	*rps11*	*ccsA*	
rpoC2	*ndhK*	*petG*	*rpl36*	*ndhD*	
rpoC1	*ndhC*	*psaJ*	*rps8*	*psaC*	
rpoB	*atpE*	*rpl33*	*rpl14*	*ndhE*	
petN	*atpB*	*rps18*	*rpl16*	*ndhG*	
psbM	*rbcL*	*rpl20*	*rps3*	*ndhI*	

Table S11. Comparison of polymorphic sites (74 CDS used in phylogenomics tree construction) between *G. mangostana* var Mesta/Manggis versus the other *Garcinia* species.

**Features**	***G. mangostana* var Mesta/Manggis versus the other *Garcinia* species**					
	G. anomala	***G. gummi-gutta***	***G. mangostana* var Thailand**	***G. oblongifolia***	***G. paucinervis***	***G. pedunculata***
Number of sites	66,117	66153	66,144	66,036	66,147	66,114
Sites with alignment gaps or missing data*	123 (0.19%)	132 (0.20%)	243 (0.37%)	180 (0.27%)	177 (0.27%)	123 (0.19%)
Invariable sites*	65,736 (99.42%)	65728 (99.36%)	65,342 (98.79%)	65,588 (99.32%)	65,325 (98.76%)	65,414 (98.94%)
Variable sites*	258 (0.39%)	293 (0.44%)	559 (0.85%)	448 (0.68%)	645 (0.98%)	577 (0.87%)

*Parentheses indicate percentage of gaps/invariable/variable sites

Table S12. List of species used for phylogenetic tree construction using ITS gene.

**Species**	**Locality/origin**	**GenBank/DDBJ* no.**
G. mangostana SAM	South America	AJ 509214
G. mangostana PM1	Peninsular Malaysia	AF 367215
G. mangostana PM2	Peninsular Malaysia	AB 110808
G. mangostana PM3	Peninsular Malaysia	AB856024*
G. mangostana MASTA	Peninsular Malaysia	AB856025*
G. mangostana JAV	Java Island	AB 110807
G. mangostana TH1	Thailand	AB110809
G. mangostana TH2	Thailand	AB110810
G. mangostana TH3	Thailand	AB110811
G. mangostana LAO	Laos	AB856023*
G. malaccensis MY1	East coast, Peninsular Malaysia	AB856027*
G. malaccensis MY2	East coast, Peninsular Malaysia	AB856028*
G. malaccensis MY3	East coast, Peninsular Malaysia	AB856029*
G. malaccensis MY4	North East, Peninsular Malaysia	AB856026*
G. malaccensis MY5	South, Peninsular Malaysia	AB856030*
G. malaccensis SUM1	Sumatra, Indonesia	AB 110805
G. malaccensis SUM2	Sumatra, Indonesia	AB 110806
G. malaccensis SBH	Sabah, Malaysia	AB856031*
G. penangiana 1	West Peninsular Malaysia	AF 367226
G. penangiana 2	East Peninsular Malaysia	AB856033*
G. diospyrifolia	West, Peninsular Malaysia	AF 367227
G. celebica	Peninsular Malaysia	AB856032*
G. atroviridis	Peninsular Malaysia	AF 367211

Asterisk denotes sequences that were submitted to DDBJ
Without asterisk sequences refers to GenBank accessions

Table S12 (continue). List of species used for phylogenetic tree construction using ITS gene.

Species	Locality/origin	GenBank
G. paucinervis CH1	China	KX421323
G. paucinervis CH2	China	KX421324
G. paucinervis CH3	China	KX421325
G. oblongifolia	China	KX421326
G. pedunculata InD1	Western Ghats, India	KP318343
G. pedunculata InD2	Western Ghats, India	KP318344
G. pedunculata InD3	Western Ghats, India	KP318345
G. pedunculata InD4	Western Ghats, India	KP318346
G. oblongifolia CH1	China	KX421327
G. oblongifolia CH2	China	KX421328
G. gummi-gutta InD1	India	KY659403
G. gummi-gutta InD2	India	KY659404
G. gummi-gutta InD3	India	KX765284
Garcinia celebica BBI1	Indonesia: Bogor Botanic Garden	LC010524
Garcinia celebica BBI2	Indonesia: Bogor Botanic Garden	LC010525
Garcinia celebica BBI3	Indonesia: Bogor Botanic Garden	LC010527
Garcinia hombroniana BBI1	Indonesia: Bogor Botanic Garden	LC010528
Garcinia hombroniana BBI2	Indonesia: Bogor Botanic Garden	LC010529
Garcinia hombroniana BBI3	Indonesia: Bogor Botanic Garden	LC010530
G. mangostana var Manggis UKM	Universiti Kebangsaan Malaysia, Bangi	OK576274
G. mangostana var Mesta UKM	Universiti Kebangsaan Malaysia, Bangi	OK576276

Table S13. Summary of different methods used for Manggis plastome assembly.

**Software**		**GetOrganelle**		**Platanus**
**Assembly Method**		**Reference-guided assembly**	***De novo* assembly**	***De novo* assembly**
Structure		Complete circular plastome	Linear circular contig with manual curation needed	5 scaffolds
No. of plastome related contig/scaffolds		1	1	5
Contig/Scaffolds name		*G. mangostana* var Manggis ref Mesta	*G. mangostana* var Manggis no ref	scaffold114, scaffold46654, scaffold418, scaffold46935, scaffold47072
Contig/scaffolds size		156,581 bp	156,663 bp	Total = 128,569 bp<br>scaffold114 = 85,517 bp<br>scaffold46654 = 22,228 bp<br>scaffold418 = 17,237 bp<br>scaffold46935 = 2,522 bp<br>scaffold47072 = 1,065 bp
Differences detected between different methods	Indel<br>Position*:<br>between 51,105-51106	Position: 51,100-51,123<br>TTCGAAAAAAAAAAAAA<br>AAAAAAT	Position: 22-45<br>TTCGAAAAAAAAAAAAAA<br>AAAAAT<br>or<br>Position: 156,603-156,627<br>TTCGAA**T**AAAAAAAAAAA<br>AAAAAAT	Position at scaffold114:<br>51,224-51,230<br>TTCGAA**T**
	SNP<br>Position*:<br>51,136	Position: 51,123-51,136<br>TCATAAAAATAAA**G**	Position: 45-58<br>TCATAAAAATAAA**G**<br>or<br>Position: 156,627-156,640<br>TCATAAAAATAAA**A**	Position at scaffold114:<br>51,266-51,279<br>TCATAAAAATAAA**G**

* Position at plastome of *G. mangostana* var Manggis ref Mesta; Red font: Indel or SNP

Table S14. CDS length comparison of *Garcinia* species before and after adjustment.

**Gene name**	***G. anomala***	***G. gummi-gutta***	***G. mangostana***		***G. oblongifolia***	***G. pedunculata***	***G. paucinervis***
			**Mesta/Manggis**	**Thailand**			
rps16 exon1	40	40	40	40	40	40	40
rps16 exon2	179	179	179	179	89/110	62	224
atpF exon 1	145	145	145	145	145	145	144/145
atpF exon 2	398	398	398	398	398	398	399/398
rpoC1 exon 1	432	432	432	432	432	432	430/432
rpoC1 exon 2	1632	1632	1635	1632	1632	1632	1634/1632
psbM	105	105	105	105	105	159/105	105
ndhK	678	678	678	678	678	762/678	678
cemA	687/717	747	714	690/726	750	690/726	726
clpP exon1	71	69/71	71	69/71	69/71	71	69/71
clpP exon2	292	291/292	292	291/292	291/292	292	291/292
clpP exon3	228	228	228	228	228	228	228
petD exon1	8	7/8	8	7/8	7/8	8	7/8
petD exon2	526	524/526	526	494/496	524/526	526	524/526
rps19	150/279	279	279	228	279	279	279

CDS length before/after adjustment

Table S14 (continue). CDS length comparison of *Garcinia* species before and after adjustment.

**Gene name**	***G. anomala***	***G. gummi-gutta***	***G. mangostana***		***G. oblongifolia***	***G. pedunculata***	***G. paucinervis***
			**Mesta/Manggis**	**Thailand**			
*rps12** exon1	114	114	114	114	114	114	114
*rps12** exon2	232	232	232	232	232	232	233/232
*rps12** exon3	26	26	26	26	26	26	25/26
ndhD	1365/1503	1503	1503	1503	1503	1557/1503	1503
ndhE	306	306	306	306	306	303/306	306
ndhA exon1	566/561	562	562	562	562	567/561	561
ndhA exon1	535/534	533	533	533	533	534	534

CDS length before/after adjustment

*The *rps12* gene is a trans-spliced gene with the 5′ end located in the LSC region and the duplicated 3′ ends in the IR region
